# CEBPD is a master transcriptional factor for hypoxia regulated proteins in glioblastoma and augments hypoxia induced invasion through extracellular matrix-integrin mediated EGFR/PI3K pathway

**DOI:** 10.1038/s41419-023-05788-y

**Published:** 2023-04-14

**Authors:** Xing-gang Mao, Xiao-yan Xue, Rui Lv, Ang Ji, Ting-yu Shi, Xiao-yan Chen, Xiao-fan Jiang, Xiang Zhang

**Affiliations:** 1 Department of Neurosurgery, Xijing Hospital, Fourth Military Medical University, Xi’an, Shaanxi Province People’s Republic of China; 2 Department of Pharmacology, School of Pharmacy, Fourth Military Medical University, Xi’an, Shaanxi Province People’s Republic of China; 3 College of Life Sciences, Northwest University, Xi’an, Shaanxi Province People’s Republic of China

**Keywords:** CNS cancer, Growth factor signalling, Extracellular signalling molecules

## Abstract

Hypoxia contributes to the initiation and progression of glioblastoma by regulating a cohort of genes called hypoxia-regulated genes (HRGs) which form a complex molecular interacting network (HRG-MINW). Transcription factors (TFs) often play central roles for MINW. The key TFs for hypoxia induced reactions were explored using proteomic analysis to identify a set of hypoxia-regulated proteins (HRPs) in GBM cells. Next, systematic TF analysis identified CEBPD as a top TF that regulates the greatest number of HRPs and HRGs. Clinical sample and public database analysis revealed that CEBPD is significantly up-regulated in GBM, high levels of CEBPD predict poor prognosis. In addition, CEBPD is highly expressed in hypoxic condition both in GBM tissue and cell lines. For molecular mechanisms, HIF1α and HIF2α can activate the CEBPD promotor. In vitro and in vivo experiments demonstrated that CEBPD knockdown impaired the invasion and growth capacity of GBM cells, especially in hypoxia condition. Next, proteomic analysis identified that CEBPD target proteins are mainly involved in the EGFR/PI3K pathway and extracellular matrix (ECM) functions. WB assays revealed that CEBPD significantly positively regulated EGFR/PI3K pathway. Chromatin immunoprecipitation (ChIP) qPCR/Seq analysis and Luciferase reporter assay demonstrated that CEBPD binds and activates the promotor of a key ECM protein FN1 (fibronectin). In addition, the interactions of FN1 and its integrin receptors are necessary for CEBPD-induced EGFR/PI3K activation by promoting EGFR phosphorylation. Furthermore, GBM sample analysis in the database corroborated that CEBPD is positively correlated with the pathway activities of EGFR/PI3K and HIF1α, especially in highly hypoxic samples. At last, HRPs are also enriched in ECM proteins, indicating that ECM activities are important components of hypoxia induced responses in GBM. In conclusion, CEPBD plays important regulatory roles in the GBM HRG-MINW as a key TF, which activates the EGFR/PI3K pathway through ECM, especially FN1, mediated EGFR phosphorylation.

## Introduction

As one of the most malignant tumors in human, effective therapeutic approach is very limited for glioblastoma (GBM) [1], mainly because of the incomplete understanding of the mechanisms of GBM development. Hypoxia played important roles in the progression of GBM in several aspects, including invasion, angiogenesis, therapeutic resistance, and stem cell maintenance [2, 3]. Hypoxia is related to the progression, as well as the initiation of GBM. Therefore, great efforts have been made to understand the mechanisms in which the hypoxia promotes the progression of GBM, and develop novel therapeutic targets against hypoxia-induced molecular responses. Hypoxia inducible factors 1A (HIF1A or HIF1α), and 2A (EPAS1, or HIF2α) are the key factors that induce gene expression levels under hypoxic condition and are important therapeutic targets [4–6]. However, HIF-targeted therapies have not been successful in patients [7] mainly because of the complicated interplay of hypoxia-related pathways and drug resistance [8]. Therefore, hypoxia-induced molecular and biological changes need to be further studied to discover potential therapeutic targets [8, 9].

An increasing number of studies has recognized that a group of interacted genes, rather than single or a few genes, contribute to biological functions. The hypoxia-induced expression changes of a cohort of genes termed as hypoxia-regulated genes (HRGs) form a complex molecular interaction network (MINW) [2]. Nonetheless, limited is known about the structures of the HRG-MINW because of the complex organization of the network. In addition, the mechanism in which hypoxia influences the biological processes of GBM has not been fully determined. Transcriptional factors (TFs) often have important roles for MINWs. Therefore, we deeply studied the key TFs that regulate the expression of HRGs and the function of HRG-MINW. Based on the systematic examination of the target of human TFs, we found that, CCAAT enhancer binding protein delta (C/EBP-delta, CEBPD) is the top TF that controls the greatest number of HRGs in GBM. In addition, CEBPD contributes to GBM invasion by activating the EGFR/PI3K pathway under hypoxia. It has been reported that CEBPD is related to hypoxia and hypoxia inducible factors [10–13], and participates in the progression of GBM [14, 15], implying its importance as a potential therapeutic target. However, CEBPD, as a central TF that regulates HRGs, has not been uncovered, especially in GBM. In addition, as a key TF for HRGs, its functional and molecular roles for GBM in hypoxia have not been fully acknowledged. Here, we demonstrated that, in GBM, CEBPD served as the top TF that controlled the HRGs, and it mostly affected the extracellular matrix (ECM)-receptor interaction and EGFR/PI3K/AKT signal pathway. Furthermore, CEBPD promoted the tumorigenesis potential of GBM through the ECM-mediated activation of the EGFR/PI3K/AKT signal pathway, especially in hypoxic condition. Specifically, FN1 (fibronectin), a key gene in ECM, is a direct target of CEBPD. The significance of EGFR/PI3K/AKT pathway on GBM has been extensively studied [16, 17], and the importance of ECM on GBM is being increasingly recognized in recent years [18–20]. Our results uncovered the intrinsic roles of CEBPD on HRGs and provided novel links between hypoxia, ECM and EGFR/PI3K/AKT pathway.

## Materials and methods

### Identification of targets of transcriptional factors (TFs) based on Algorithm for the Reconstruction of Accurate Cellular Networks (ARACNe)

ARACNe was used for inferring transcriptional targets for each TF based on several independent GBM expression datasets, as described previously. A threshold of 10^−7^ was used to obtain candidate genes that may be regulated by a TF, directly or indirectly. Totally five GBM datasets were used to infer several lists of TF-regulated targets, as has been described previously [2] (a unified GBM data from TCGA [21], a validation GBM data from Verhaak et al. [21], the GBM data from Rembrendt (GSE68848) [22], a GBM dataset from Lee et al. (GSE13041) [23], and a GBM data from Gravendeel et al. (GSE16011) [24], which contained 197, 260, 228, 191, and 159 samples, respectively). Then target genes that were identified in at least one dataset were used as the targets for each TF.

### Glioma samples

Glioma or normal brain samples used for real-time quantitative (q)PCR and Western blot were provided by the Department of Neurosurgery, Xijing Hospital, the Fourth Military Medical University, as described previously [25], including 31 GBM, 14 grade II astrocytomas (A), 15 grade III anaplastic astrocytomas (AA), and 5 normal brain samples derived from brain lobectomy from patients with cerebral trauma. A tissue chip containing 5 normal brain, 30 A, 20 AA, and 50 GBM were established and used for immunohistochemistry. Tumors were classified according to WHO classification. The study protocol was approved by the institutional review board of Xijing Hospital of the Fourth Military Medical University, and written informed consent was obtained from patients.

### Data accessibility

GBM datasets, including REMBRANDT data (GSE68848), GSE13041, GSE16011, and hypoxia-related datasets (GSE32100, GSE45301, GSE27523) can be downloaded from GEO website (https://www.ncbi.nlm.nih.gov/gds/). The glioblastoma atlas project (GAP) data were downloaded from http://glioblastoma.alleninstitute.org/ [26]. The unified GBM data from TCGA, and the validation GBM data were originated from the research of Verhaak et al. [21]. CGGA data can be downloaded from http://www.cgga.org.cn/download.jsp. Prognosis analysis of TCGA and CGGA data were performed in http://xena.ucsc.edu/ and the http://www.cgga.org.cn/analyse/RNA-data.jsp. Data for Pathway and expression analysis were downloaded from TCGA data hub https://xenabrowser.net/hub/.

### Culture of glioma cells

Human glioblastoma cells U251 and U87 cells (U251 and U87) were cultured in 10% serum medium. U87 cells were purchased from ATCC, and U251 cells were purchased from National Collection of Authenticated Cell Cultures of Chinese Academy of Sciences. Both cell lines were authenticated with short tandem repeat (STR) Profiles. To induce hypoxia, cells were cultured in a sealed Modular Incubator Chamber (Billups-Rothenberg Inc., Del Mar, CA) flushed with 1% O_2_, 5% CO_2_, and 94% N_2_ at 37 °C for 24 h.

### Tandem mass tags (TMT) quantitative proteomics

TMT assays were performed triplicate or duplicates in each group. A protein was considered as a differential one when the difference ratio is 1.2 times or more, and *P* < 0.05. Proteins that are significantly up-regulated in hypoxia condition were termed as hypoxia-regulated proteins (HRPs). Cell samples were harvested and SDT lysis buffer (4% SDS, 100 mM DTT, 150 mM Tris-HCl pH 8.0) were added, which was sonicated and then boiled for 15 min, and centrifuged at 14,000 × *g* for 40 min. Then the supernatant was quantified with the BCA Protein Assay Kit (P0012, Beyotime), and stored at −80 °C until further analysis. For each sample, proteins were mixed with 5X loading buffer, boiled for 5 min, and then separated on 12.5% SDS-PAGE gel. Protein bands were visualized by Coomassie Blue R-250 staining. For each sample, 200 μg proteins were incorporated into 30 μl SDT buffer. Repeated ultrafiltration (Sartorius, 30 kD) by UA buffer (8 M Urea, 150 mM Tris-HCl pH 8.5) was performed to remove the detergent, DTT and other low-molecular-weight components. To block reduced cysteine residues, 100 μl iodoacetamide (100 mM IAA in UA buffer) was added and the samples were incubated for 30 min in darkness. The filters were washed with UA buffer and 0.1 M TEAB buffer. At last, the protein suspensions were digested with 4 μg trypsin (Promega) in 40 μl 0.1 M TEAB buffer overnight at 37 °C, and the resulting peptides were collected as a filtrate. The peptide content was estimated by UV light spectral density at 280 nm.

Next, for each sample, 100 μg peptide mixture was labeled using TMT reagent according to the manufacturer’s instructions (Thermo Fisher Scientific). Then the TMT-labeled peptides were fractionated by reversed-phase chromatography using the Agilent 1260 infinity II HPLC. The collected fractions were dried down via vacuum centrifugation at 45 °C. Subsequently, LC-MS/MS analysis was performed on a Q Exactive Plus mass spectrometer (Thermo Fisher Scientific) that was coupled to Easy nLC (Thermo Fisher Scientific). MS/MS spectra were searched using MASCOT engine (Matrix Science, London, UK; version 2.6) embedded into Proteome Discoverer 2.1.

### Quantitative real-time PCR

RNA was extracted from cultured cells and brain tumor tissues using Trizol Reagent (Invitrogen). Extracted RNA was reverse transcribed into cDNA and qPCR analysis was performed on an ABI7700 system using SYBR Green PCR Core Reagents in 20 μL reactions (Applied Biosystems, Warrington, UK). Water instead of template was used as the negative control. Primers used for qPCR analysis were as follows: CEBPD forward: CCCCGCCATGTACGACGAC, CEBPD reverse: CCCGCCTTGTGATTGCTGT; EGFR forward: CCCACTCATGCTCTACAACCC; EGFR reverse: TCGCACTTCTTACACTTGCGG; FN1 Forward: GCTGCACATTGCCTGTTCTG, FN1Reverse: TCCTACAGTATTGCGGGCCA; β-actin forward: GGCACCCAGCACAATGAA, β-actin reverse: TAGAAGCATTTGCGGTGG. All samples were assayed in triplicate and the relative amount of target transcripts normalized to the number of human β-actin transcripts in the same sample. Relative fold changes were calculated using the ΔΔCt method with the threshold cycle values of each sample.

### Western blotting

Western blotting (WB) was performed as described [25]. Antibodies were used as per the manufacturer’s instructions and were as follows: CEBPD (Abcam, ab245214; or Invitrogen, PA5-30262); HIF1α (Abcam, #ab179483): EGFR (Cell Signaling Technology, #4267); p-EGFR (Tyr1173) (Cell Signaling Technology, #4407), ERK1/2 (Cell Signaling Technology, #4695), p-ERK1/2 (thr202/tyr204) (Cell Signaling Technology, #4370), AKT (Cell Signaling Technology, #4685), p-AKT (Ser473) (Cell Signaling Technology, #4060), mTOR (Wanleibio, Shenyang, China, WL02477), p-mTOR (Ser2448) (Wanleibio, WL03694), STAT3 (Cell Signaling Technology, #9139), p-STAT3 (Ser 727) (Cell Signaling Technology, #9134), FN1 (Proteintech, 15613-1-AP), COL1A1 (Wanleibio, WL0088), TNC (Proteintech, 67710-1-Ig), β-actin (Wanleibio, WL01372). All WBs were performed in triplicate, and quantification of protein expression levels were performed by using the ImageJ software (National Institutes of Health, Bethesda, MD).

### Immunohistochemistry

Paraffin-embedded, 1-mm formalin fixed tissue sections were mounted on microscope slides and processed as previously described. Immunohistochemical staining of anti-human CEBPD (1:200, Biorbyt, orb213725) was performed on tissue sections. The sections were treated with a heat-induced epitope retrieval technique using a citrate buffer at pH 6.0. The sections were then blocked for endogenous peroxidase and biotin before incubation with primary antibodies for 3 h at room temperature. The Elite Vector Stain ABC System (Vector Laboratories) was used as the detection system and diaminobenzidine as the chromogen. Nuclei were counterstained with hematoxylin. The immunoreactivity score of CEBPD was determined by multiplication of the values of the percentage of CEBPD positive cells (0: 1%, 1: 1–25%, 2: 26 –50%, 3: 51–75%, 4: 75%) and the values for CEBPD staining intensity (0: no staining, 1: weak staining, 2: moderate staining, 3: strong staining), as described previously [27].

### Identification of hypoxia-regulated genes (HRGs)

To get a reliable HRGs set, we used hypoxia-regulated genes identified from 5 datasets, as described in our previous study [2], including two U87 cells data (GSE32100 [28] and GSE45301 [29]), 1 glioma stem-like cells (GSC) data (GSE32100 [28]), 1 serum cultured GSC cell data (GSE32100 [28]), and one LN229 cell line data (GSE27523 [30]). The genes which are statistically significantly up-regulated in hypoxia (*p* < 0.05) more than 1.5 folds are chosen as candidate HRGs in GBM. By taking advantages of the general hyper-geometric distribution (GHG) [31], a statistically reliable HRGs (SR-HRGs) were obtained by using genes that are overlapped in at least 3 gene sets in the above 5 results. The GHG was used because if we use the genes overlapped in at least 5 or 4 datasets, then we only get a few genes and would lose too many HRGs, while if we use genes overlapped in too few sets then the resulted HRGs would contain too many false positive genes [31, 32].

### Key transcription factor (TF) identification

To get TFs that have regulatory impact on more genes in the HRGs, we systemically analyzed the human TFs, and calculated the number of genes belong to the HRPs, HRGs, or genes specifically expressed in hypoxic pseudopalisading cells around necrosis (PANSGs) in GAP dataset, regulated by each TF (N_TF_HRPs, or N_TF_HRGs, N_TF_PANSGs). Then the TFs are ranked according to their N_TF_HRPs, N_TF_HRGs, or N_TF_PANSGs values. First, for all human TFs [33], the potential regulatory target genes for each TF in GBM were defined as inferred targets based on mutual information [2, 34], or combined with a TF target database [35]. Then the TF-regulated genes belong to the HRGs were selected, and ranked according to their N_TF-HRPs, N_TF_HRGs, or N_TF_PANSGs. Tops TFs were considered as key TFs that would have important regulatory impact on hypoxia-induced genes. For statistical hypothesis testing, we used random gene list (containing the same number of genes of the HRPs or HRGs), and then used the same procedure to get TF ranks. Then the ranks of CEBPD or CEBPB were analyzed for 1000 random repeats, to calculate the distribution of rank position of CEBPD or CEBPB.

### shRNA lentivirus infection

shRNA lentivirus particles targeting CEBPD (shCEBPD-KD1 and shCEBPD-KD2, or Abbreviated as shKD1 and shKD2, if not confused) and a scramble non-targeting shRNA (shCtrl) were purchased from GENECHEM (SHANGHAI GENECHEM CO., LTD). Cells were infected with shRNA lentiviruses according to the manufacturer’s protocol. Briefly, U87 and U251 cells were dissociated into single cells with Accutase and gentle trituration, and then incubated with lentivirus for 24 h. After approximately 48 h, 2 μg/ml puromycin was used to select infected cells. shRNA target sequences for CEBPD were shCEBPD-KD1: CGACCTCTTCAACAGCAAT; shCEBPD-KD2: GGGACATAGGAGCGCAAAGAA.

### Rescue experiments after CEBPD knockdown

For integrin receptor rescue assay, β1 integrin agonist pyrintegrin (1 μM; MCE, Cat#: HY13306), which can activate the FN1 receptor α5β1 integrin [36], were used to treat U87-CEBPD KD cells for 48 h. In addition, a recombinant FN1 protein (rFN1, 5 μg/ml; MCE, Cat#: HY-P73063) was used to treat the U87-CEBPD KD cells for 48 h.

### CCK-8 assay

U87 and U251 cells were cultured in 96-well plates, and the IC50 values were calculated by performing CCK-8 assays in investigated time points. Before detection, 10 μL of reagent was added to the well plate. After incubation in the incubator for 1 h, the absorbance at 490 nm was detected using a Bio-Rad iMark plate reader (Bio-Rad Laboratories, Inc). The detection was performed in investigated time points. Triplicate tests were performed in each time point, and growth curves were plotted.

### Transwell invasion assay

Transwell invasion assays were performed on Corning® BioCoat™ Matrigel® Invasion Chambers. After 24 hours incubation, the cells remaining on top chamber were removed with a cotton-tipped applicator, and cells on the bottom of the membrane were fixed with 80% methanol and counterstained with hematoxylin. Cells were photographed at high power (200X) and three separate high-power fields were counted.

### In vivo flank and intracranial xenograft tumors

Intracranial xenograft experiments were performed as described previously. Briefly, isolated cells were resuspended in 10 mL PBS and stereotactically injected into the right striatum of the brains (10^5^ cells) or into the flank (10^6^ cells) of nude mice (6–8 weeks old; *n* = 5 for each group; Center of Experimental Animals, Fourth Military Medical University), following administration of general anesthesia. Coordinates for stereotactic injections into the adult mice were 2 mm to the right of the midline, 0.5 mm anterior to the coronal suture, and 3 mm deep. For the flank xenografts, tumor volumes were investigated at each time point, and the mice were sacrificed 8 weeks later and examined for tumor formation in the brains. For the intracranial xenograft Tumors, the mice were sacrificed at about 20 days later when there are symptoms. ImageJ software (National Institutes of Health, Bethesda, MD) was used for area calculation and invasive fingers recognition. Briefly, the tumor areas were delineated by using the following processes: red channel split (because the red channel had the best contrast), image binarization, fill holes, and remove small noises. The same processes and parameters were used for all images. All animal handling during the experiments was in strict accordance with the Animal Experiments guidelines in force at the Fourth Military Medical University.

### Chromatin immunoprecipitation (ChIP) qPCR and ChIP-sequencing (ChIP-seq)

Cells were cross-linked with 1% formaldehyde solution, neutralized by 1.25 M glycine, then lysed and sonicated. The sonicated chromatin was precleared with ChIP Grade protein A/G plus Agarose for 1 h. Then the lysates were incubated at 4 °C overnight with the target-specific antibody HIF1A (Abcam, #ab51608) or CEBPD (Invitrogen, PA5-30262), or negative control of normal Rabbit and Antibody. DNA was eluted from immunoprecipitated complexes, reverse cross-linked, and purified. High-quality ChIP DNA were used for qPCR, or sequenced on HiSeq X ten PE150NovaSeq 6000 in GENECHEM CO., LTD. The primers used for ChIP PCR analysis were as follows: CEBPD promotor fragment (303 bp) forward: AGGAGAGCAGCGAGAACCG, reverse: CCTCGGGGTTGGGAGTGAAA. FN1 promotor fragment (255 bp) forward: AGCAATTGTGCCTGTGTTTATCT; reverse: CTTCGGCCCCTTTCTCACAT. EGFR promotor fragment (300 bp) forward: CCTGCTCGGCCATACAGTTT, reverse: GCCTTCATAGTACGGCTTGT. The Chip-qPCR were repeated in triplicate. The ChIP-seq results were visualized with Integrative genomics viewer [37]. The number of CEBPD binding sites in the promotor region (0–3 kb, 0–2 kb, and 0–1 kb) of interested genes were analyzed to find target genes that can be transcriptionally regulated by CEBPD.

### Luciferase reporter assay

Luciferase reporter vectors were performed with Dual-Luciferase® Reporter Assay System purchased from GENECHEM. The FN1 promotor and CEBPD sequence were synthetized. The HIF1α, EPAS1 sequences, and CEBPD promotor region were amplified using PCR. The primers were as follows: HIF1A-P1: ATGGAGGGCGCCGGCGGCGCGAACG; HIF1A-P2: GTTAACTTGATCCAAAGCTCTG; EPAS1-P1: CGCCACCATGACAGCTGACAAGGAGAAGAAAAG; EPAS1-P2: GGTGGCCTGGTCCAGGGCTCTGAG; CEBPD promotor P1: AGTGATTAGGGAGGGCTTTAATAG; CEBPD promotor P2: GGCGGCGTCGGGCCGGGCTCTGC. Then the cDNA fragments of each promoter were inserted into pGL3-Basic vectors, and the sequences of each TF were inserted into pRL-TK vectors. U87 cells were co-transfected with the above pGL3-Basic (containing promotor sequence or blank control) and pRL-TK vectors (containing TF sequence, TF shRNA sequence, or blank control). Luciferase activities were measured with the Dual-Luciferase Reporter Assay System (Promega) 24 h after transfection. Data were normalized firefly luciferase activity to Renilla luciferase activity for each set of triplicate samples. Then the results were compared to vector only control groups. All experiments were performed at least three times independently.

### Association analysis between gene expression level and pathway activity levels

The pathway activity was determined as described previously [38], and the pathway activity data were obtained from TCGA data hub (https://xenabrowser.net/datapages/). The association between gene expression and pathway activity level was examined with Pearson Correlation.

### High and low hypoxia GBM samples (HHS and LHS) analysis

In summary, the expression level of each gene in HRGs was ranked in all GBM samples, and the rank index was given for each sample. Then an integrated hypoxia-score for each sample was defined by calculating the sum of all HRG genes’ rank index.

High and low hypoxia GBM samples (HHS and LHS) were determined by their integrated hypoxia scores (IHS) for each sample. To give an IHS, we calculated the rank position of each gene belong to the HRGs. Then the rank positions for all genes belong to the HRGs were summed to get an IHS for the sample. Next, all of the TCGA GBM samples were ranked according to their IHSs. HHSs were defined as the top 45% samples ranked according to their IHSs, and LHS were the bottom 45% samples. To determine the impact of hypoxia, the expression or pathway activity levels were obtained from HHSs and LHSs. To determine the impact of hypoxia on CEBPD’s function, the association between CEBPD expression level and pathway activities (HIF1α pathway, or EGFR/PI3K/AKT related pathways) were analyzed in HHSs and LHSs respectively.

### Statistical analysis

Statistical analysis was performed using Two-tailed unpaired Student’s t-test or one-way ANOVA, as appropriate. *P* < 0.05 was considered statistically significant. The correlation between two variables were analyzed using Pearson’s correlation. Log-rank test and Kaplan–Meier analysis were used to assess the survival difference. Data in figures were displayed as mean ± SD. Statistical analysis was performed with SPSS v.16.0.

## Results

### Identification of CEBPD as a master transcription factor for hypoxia induced genes in glioblastoma in both protein and mRNA levels

**Fig. 1:**
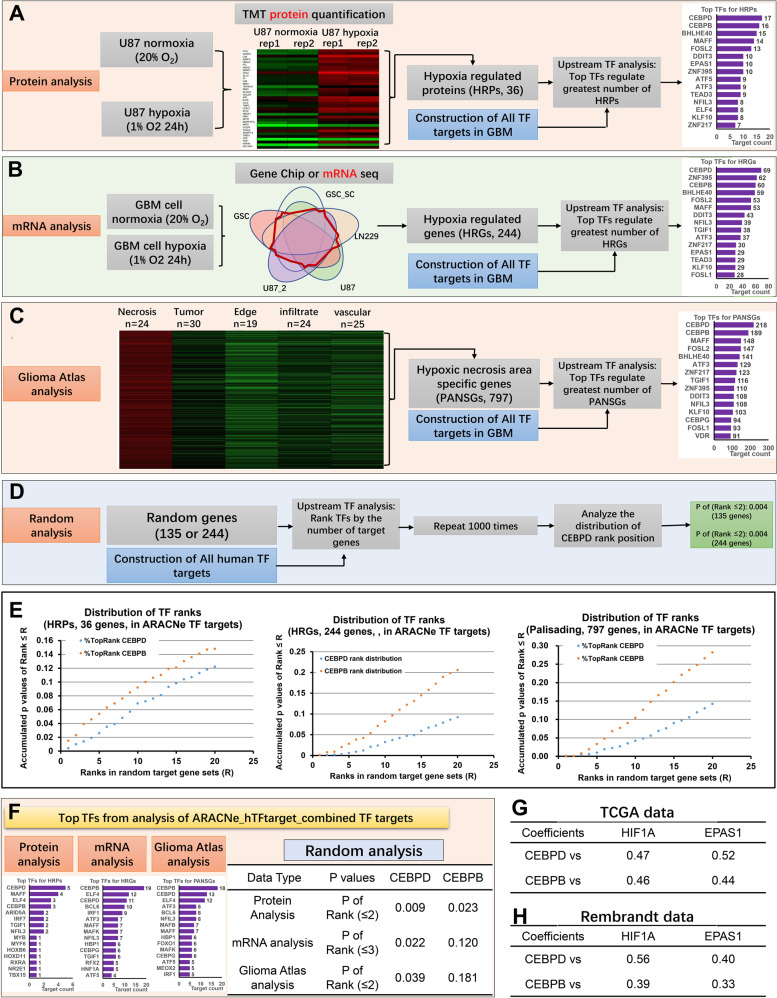
Procedures of identifying key transcription factors (TFs) of the hypoxia-regulated genes in GBM. **A** identification of key TFs from hypoxia-regulated proteins (HRPs). Quantitative TMT proteomic analysis identified significantly up-regulated proteins (HRPs) for at least 1.2-fold change under hypoxia. Then systemic TF-targets analysis identified top TFs which regulated the most number of targets belong to HRPs for all human TFs. The targets of each TF were identified with the mutual information-based ARACNe algorithms, as described previously [2]. **B** Similar procedure was performed in hypoxia-regulated genes (HRGs) in mRNA level, by using the statistically-reliable HRGs (SR-HRGs), which are genes overlapped in at least 3 sets from 5 hypoxia-induced gene sets. Then top TFs regulating the most number of targets belong to SR-HRGs were identified. **C** Similar procedure was further performed in pseudopalisading cells around necrosis (PAN) specific genes (PANSGs) in GBM tissues from glioblastoma atlas data [26]. PANSGs were those that are specifically significantly highly expressed in PAN area (*p* < 0.05), and with an averaged fold change greater than 2, compared to the other areas, including leading edge (LE), infiltrating tumor (IT), cellular tumor (CT), and microvascular proliferation (MVP). Then top TFs regulating the most number of targets that are specifically highly expressed in PAN area were identified. **D** Top TF analysis for Random target genes. Certain number (the same number of genes of the HRPs, HRGs, and PANSGs) of random genes, which are randomly selected from the corresponding background genome (all genes of the high-throughput platform), were produced to analyze the top TFs that regulate the random genes. Totally 1000 random gene sets were analyzed to produce a rank position distribution for each TF. **E** Rank position distributions of CEBPD and CEBPB in random target gene set analysis. Random targets with 36, 244, or 797 genes, were analyzed, corresponding to the gene numbers of HRPs, HRGs, and PANSGs, respectively. Data were obtained by repeating 1000 times. Rank distributions were analyzed for ARACNe-based TF targets. **F** Top TF analysis and rank distribution analysis of CEBPD and CEBPB for HRPs, HRGs, and PANSGs, where the targets of each TF were defined by the combined targets of ARACNe and hTFtarget-based targets [35] (ARACNe_hTFtarget_combined). **G**, **H** Correlations between top TFs (CEBPD and CEBPB) and HIFs (HIF1α and HIF2α/EPAS1) in TCGA (**G**) and Rembrandt (**H**) GBM databases.

Protein level changes under hypoxia condition were investigated using proteomic analysis with tandem mass tags (TMT) techniques. U87 glioma cells were used to obtain comprehensive hypoxia-regulated proteins (HRP), which are up-regulated under hypoxia condition (1% O_2_). As a result, a list of 498 proteins that were significantly up-regulated in hypoxia were obtained, including 36 proteins that are up-regulated by 1.2-folds or higher (Supplementary Table S1). Top transcription factor (TF) analysis was performed to determine the master up-stream TFs that regulate HRPs in GBM by systematically analyzing the target genes of all the human TFs. First, by using Algorithm for the Reconstruction of Accurate Cellular Networks (ARACNe) [25, 34], genome-wide target genes for each TF were identified in GBM context (1219 TFs in total) based on five independent GBM expression datasets for all the human TFs [33] (Supplementary Table S2). Next, the TFs were ranked according to the number of their target genes that belong to the HRPs. As a result, CEBPD and CEBPB were identified as the top two ones, and the top TFs include EPAS1 (HIF2α, rank 7) and ZNF217 (rank 16), among others (Fig. 1A). The top TFs containing HIF2α supported the reliability of the HRPs and the inferred TF-targets results.

Our results were validated using statistically significant reliable hypoxia-regulated genes (SR-HRGs) in mRNA levels by combining HRGs from five datasets, based on a previous study [2]. Totally, 244 genes that are overlapped in at least three of the five HRG sets were ultimately identified as SR-HRGs according to the general hyper-geometric distribution (GHG) analysis [2, 31, 32]. Top TF analysis demonstrated that CEBPD was the top TF, and the following TFs include CEBPB (rank 3), ZNF217 (rank 11), HIF2α (rank 12), and HIF1α (rank 29) (Fig. 1B).

The above conclusion was further validated in tumor samples by studying the glioblastoma atlas project (GAP) [26] to examine the genomic expression profiles in different areas in GBM tumors. In the GAP data, the pseudopalisading cells around necrosis (PAN) were in the hypoxia area, as supported by the results that most hypoxic marker genes, such as VEGFA, CA9, CA12, and LOX, were highly expressed in this area (Supplementary Figure S1). Totally, 797 PAN specific genes (PANSGs) were specifically and more highly expressed in the PAN area for at least two folds compared with that in to the other areas. For these PANSGs, 18 and 104 genes are among the HRPs (accounting for 50.00% HRPs) and HRGs (accounting for 42.62% HRGs), respectively. Interestingly, the top TF analysis also revealed that CEBPD and CEBPB are the top two TFs for these genes (Fig. 1C).

Notably, for the three kinds of hypoxia-regulated targets (HRTs, including HRPs, SR-HRGs, and PANSGs), 38.89% and 50.00% HRPs were among the HRGs and PANSGs, respectively. Based on the top TF analysis, CEBPD targets in the three kinds of HRTs also exhibited apparent overlapping, in which 35.29% and 64.71% CEBPD targets in HRPs are among that in CEBPD targets in HRGs and PANSGs, respectively.

Interestingly, analysis of all the three kinds of HRTs lead to the same conclusion that CEBPD is the top TF. Then, we examined whether this would be a biased results introduced by the algorithm. Accordingly, top TF analysis was performed on random target gene sets containing the same number of corresponding HRTs (Fig. 1D). Based on the examination of the rank position distribution of each TF in repeated random analysis, the probabilities of CEBPD with a rank position smaller than 5 is very low (*P* < 0.05; Fig. 1E) in all of the three HRTs, while those of CEBPB are higher than that of CEBPD, because CEBPB has more target genes in the entire genome (Fig. 1E). Therefore, it is an intrinsic nature for CEBPD as a top TF for HRTs.

The above conclusion was further validated by establishing more accurate TF targets by combining the ARACNe inferred TF targets and the TF target database (ARACNe_hTFtarget_combined, Supplementary Table S3). In the TF target database, the targets of each TF were identified by integrating huge human TF target resources and epigenetic modification information [35]. The top TF analysis also revealed that CEBPD was the top TF that regulated the highest number of HRPs, SR-HRGs, or PANSGs (Fig. 1F). In addition, although both the top two TFs CEBPD and CEBPB are positively correlated with HIFs (HIF1α and HIF2α) in the GBM datasets, the coefficients between CEBPD and HIFs are higher than that of CEBPB (Fig. 1G, H). Therefore, CEBPD was used for further studies.

### CEBPD is highly expressed in GBM samples and increased along with tumor grade

**Fig. 2:**
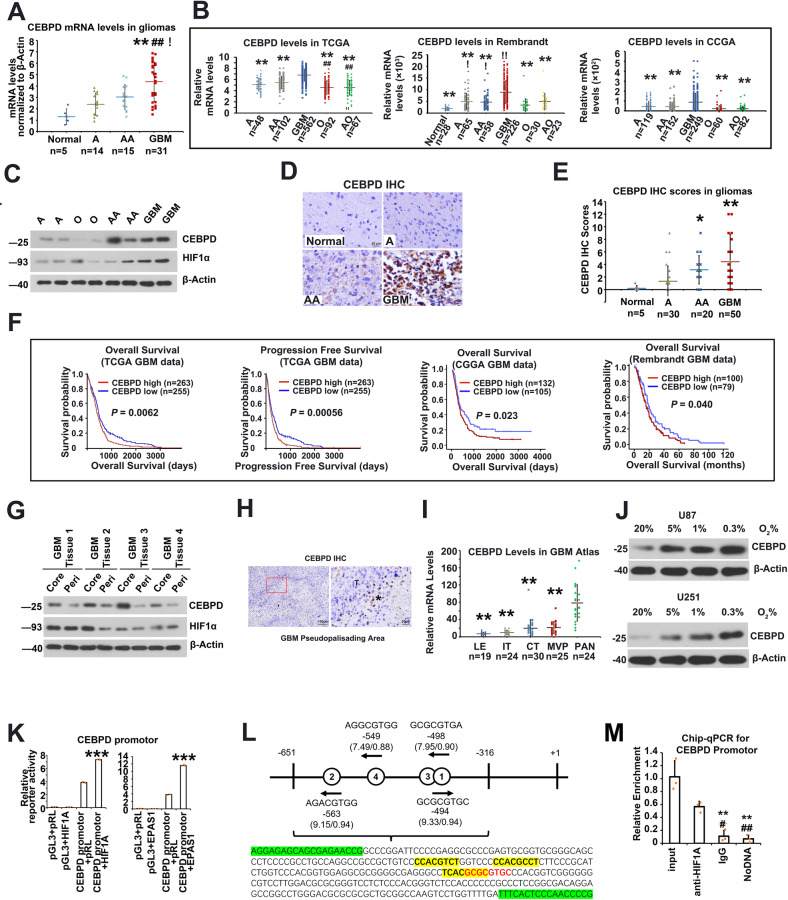
CEBPD is highly expressed in GBM and is up-regulated under hypoxia. **A** The mRNA levels of CEBPD, as revealed by qPCR, in a cohort of glioma samples with different grades (**p < 0.001 compared to Normal brain samples; ##*p* < 0.001 compared to grade 2 astrocytoma (A); !*p* < 0.05 compared to grade 3 anaplastic astrocytoma (AA). **B** Expression of CEBPD mRNA levels in different grades of gliomas in TCGA, Rembrandt, and CGGA datasets. (**p < 0.001 compared to Grade 4 GBM; ##*p* < 0.001 compared to Grade 3 AA; !*p* < 0.05, !!*p* < 0.001, compared to Normal brain samples.) **C** Representative WB results showing protein levels of CEBPD and HIF1α in different grade gliomas. **D**, **E** Representative IHC staining and quantitative staining scores of CEBPD in glioma samples (*p < 0.05, **p < 0.001, compared to grade 2 astrocytoma). **F** Survival analysis and progression-free analysis of GBM patients in different databases (TCGA, CGGA, and Rembrandt data). **G** WB results showing CEBPD protein levels in core hypoxic and peripheral less hypoxic areas in GBM samples (*n* = 3 independent experiments). **H** IHC staining showing CEBPD stain in hypoxic pseudo-palisading areas in GBM tissues; *, T, indicated pseudo-palisading areas and tumor area, respectively. **I** CEBPD mRNA levels in different areas in GBM tissues, including leading edge (LE), infiltrating tumor (IT), cellular tumor (CT), pseudopalisading cells around necrosis (PAN), and microvascular proliferation (MVP) areas, in the glioblastoma atlas data [26]; **p < 0.001, compared to PAN. **J** WB results showing CEBPD protein levels in GBM U87 and U251 cells under hypoxia condition. **K** Luciferase reporter assay showing bind and activation of HIF1α and HIF2α to the CEBPD promotor region (***p < 0.0001 compared to CEBPD-promotor-HIF-NC group). (pGL3: pGL3-Basic vector containing blank control; CEBPD promotor: pGL3-Basic vector containing CEBPD promoter sequence; pRL: pRL-TK vector containing blank control; HIF1A or EPAS1: pRL-TK vector containing HIF1A or EPAS1 TF sequence). **L** The DNA fragment with the highly reliable HIF1A or EPAS1 binding sites (Hypoxia-responsive Element, HRE) in the CEBPD promoter, as predicted by the JASPAR database [43]. There are 4 highly reliable HREs (bold letters) in the fragment. For each HRE, the sequence, location, and the binding scores (expressed as score/relative score) were labeled; red letters represent positive strand, while yellow shaded letters represent negative strand. Greed shaded letters represent sequences for primers. **M** Chip-qPCR showing that HIF1A bind to the CEBPD promoter region shown in (**L**). A grade 2 astrocytoma; O grade 2 oligodendroglioma; AA grade 3 astrocytoma; GBM grade 4 glioblastoma; peri peripheral.

The potential biological functions of CEBPD for glioma was explored. First, the mRNA levels of CEBPD in glioma tissues was investigated by qPCR, including 5 normal brain samples, 14 astrocytomas (A, grade II), 15 anaplastic astrocytomas (AA, grade III), and 31 GBMs. As a result, CEBPD mRNA levels increased along with tumor grades and became significantly overexpressed in GBMs than in normal and low-grade gliomas (LGG; *P* < 0.05, Tukey HSD ANOVA; Fig. 2A). Therefore, CEBPD is specifically activated in GBM. In addition, the analysis of the Repository of Molecular Brain Neoplasia Data (REMBRANDT) [22], The Cancer Genome Atlas (TCGA) data combing GBM and LGG expression [39], and the Chinese Glioma Genome Atlas (CGGA) databases [40] supports that CEBPD is more highly expressed in GBM than in normal and LGGs (Fig. 2B).

The protein levels of CEBPD in glioma tissues were further investigated. Western blot (WB) analysis in glioma tissues, including 2 grade II A, 2 grade II oligodendroglioma (O), 2 grade III AA, and 2 GBM samples, revealed a trend that CEBPD increased along with tumor grade (Fig. 2C).

The increased expression of CEBPD protein levels in GBMs was confirmed by performing immunohistochemistry (IHC) in a tissue chip, including 5 normal brain samples, 30 grade II A, 20 grade III AA, and 50 GBM samples, and the IHC intensity was semi-quantified (Fig. 2D). No cell staining of CEBPD was observed in normal brain tissue besides background staining (Fig. 2D). Semi-quantitative analysis revealed that CEBPD staining scores increased along with tumor grade and is significantly higher in GBM than in normal brain and LGGs (Fig. 2E).

The clinical significance of high levels of CEBPD in GBM was investigated demonstrating that the high expression of CEBPD predicted the poor prognosis of patients with GBM and LGGs (Fig. 2F; Supplementary Figure S2) in different datasets, and is an independent risk factor for patients with GBM (Supplementary Table S4).

### CEBPD is highly expressed in hypoxic condition in GBM samples and cells

As a master TF for hypoxia-induced targets, the influence of hypoxia on CEBPD expression was investigated. First, a significant positive correlation was observed between CEBPD and HIFs (HIF1α and HIF2α) in GBMs (Fig. 1G, H; Supplementary Figure S3). Next, the expression of CEBPD was compared in 6 GBM tissues derived from core necrosis area (CA) or peripheral less-hypoxic area (PA). CEBPD is more highly expressed in CA than in PA in both mRNA and protein levels (Fig. 2G, Supplementary Figure S4). Moreover, the HIF1α protein level was more highly expressed in GBM than in LGGs (Fig. 2C), and could be more highly expressed in CA than in PA (Fig. 2G, Supplementary Figure S4). Furthermore, pseudopalisades areas are more hypoxic in GBM [41, 42], and the IHC staining revealed that CEBPD is more strongly expressed in cells in the pseudopalisades area than in other areas (Fig. 2H). Based on the investigation of GAP data, CEBPD is highly expressed in the hypoxic PAN area than in the other areas (Fig. 2I). Therefore, CEBPD is more highly expressed in hypoxic condition in GBM tissues.

Next, the influence of hypoxia on CEBPD expression was investigated in GBM cells. Accordingly, U87 and U251 glioma cell lines were cultured under different oxygen levels (20%, 5%, 1%, or 0.3% O_2_ levels) for 24 h. Then, q-PCR and WB assays revealed that CEBPD was up-regulated with the decrease in oxygen levels in both cell lines, in protein and mRNA levels (Fig. 2J, Supplementary Figure S5).

The ability of HIF1α and HIF2α to bind to and activate the promotor of CEBPD was determined. Luciferase reporter assay, including a 2000 bp up-stream promoter region of CEBPD, revealed that both HIF1α and HIF2α significantly activated the promoter region of CEBPD compared with the control (Fig. 2K). In addition, chromatin immunoprecipitation (Chip)-qPCR confirmed that HIF1α could bind to the CEBPD promotor region with a high score predicted with JASPAR database [43] (Fig. 2L, M). Therefore, CEBPD can be transcriptionally regulated by HIF1α or HIF2α directly.

### CEBPD is important for the tumorigenic potential of GBM cells in vitro, and contributes to hypoxia enhanced invasion capacity

**Fig. 3:**
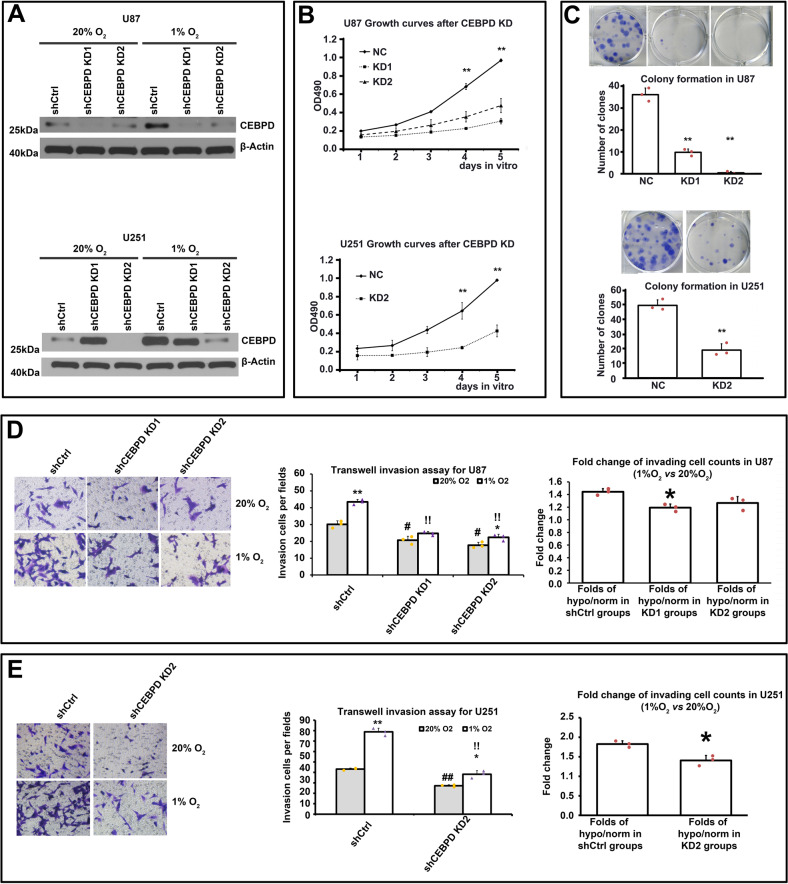
knockdown of CEBPD inhibited invasion capacity of GBM cells. **A** WB assays showing proteins levels of CEBPD after shCEBPD lentiviruses infection in U87 (up panel) and U251 (bottom panel) cells, in normoxia (20% O_2_) or hypoxia (1% O_2_) conditions. **B** In vitro CCK8 assays showing the effect of CEBPD knockdown on growth curves for U87 (up panel) and U251 cells (bottom panel). **C** In vitro colony formation assays of U87 (up panel) and U251 (bottom panel) cells with shCtrl or shCEBPD infection. **D**, **E** Transwell invasion assays of U87 (**D**) and U251 (**E**) cells infected with shCtrl or shCEBPD lentiviruses in normoxia (20% O_2_) or hypoxia (1% O_2_) conditions. **p < 0.001, *p < 0.05, compared with corresponding normoxia (20% O_2_) group; #*p* < 0.05 compared with shCtrl group in normoxia (20% O_2_); !*p* < 0.05 compared with shCtrl group in hypoxia (1% O_2_). Right images in (**D**, **E**): Fold increase of number of hypoxia induced invasion cells after shCtrl or shCEBPD treatment in U87 (**D**, right image) and U251 (**E**, right imag) cells. Repeated data for each group were obtained by calculating the ratio of each hypoxia data to the averaged value of normoxia data. *p < 0.05, compared with shCtrl group. Experiments were repeated in triplicate.

Then, the use of CEBPD for the tumorigenic potential of GBM cells in vitro was determined. U87 and U251 cells were knocked-down with CEBPD shRNA lentivirus (Fig. 3A). Two shRNA sequences, namely, shCEBPD-KD1 and shCEBPD-KD2, with significant CEBPD knockdown efficiency were developed. The knockdown efficiencies of the lentiviruses were validated in both mRNA (Supplementary Figure S6) and protein levels in both U87 and U251 cells (Fig. 3A). Considering that shCEBPD-KD1 shRNA did not exhibit validated knockdown effect in U251 in normoxic condition (Fig. 3A), it was not used for further experiments in U251 cells.

Then, growth curve experiments revealed that compared with the scramble-shRNA control group (shCtrl), the growth of GBM cells was significantly inhibited in shCEBPD-KD groups in both cell lines (Fig. 3B). In addition, shCEBPD-KD inhibited the colony formation of the two GBM cell lines (Fig. 3C).

Next, trans-well invasion assays were performed to investigate the influence of CEBPD knockdown on the invasion capacity of GBM cells, especially in hypoxia. First, shCEBPD-KD significantly decreased the invasion capacity of both U87 and U251 cells (Fig. 3D, E) either under normoxic or hypoxic condition. Notably, hypoxia induced an increase in the number of cells invading across the transwell (Fig. 3D, E), which supports previous findings [4]. Further analysis revealed that shCEBPD-KD attenuated hypoxia enhanced invasion, because the fold change of hypoxia-induced invading cell numbers in the shCtrl group are significantly greater than that in shCEBPD-KD groups in both cell lines (Fig. 3D, E). Therefore, CEBPD contributes to hypoxia-promoted invasion of GBM cells.

### CEBPD promotes the tumorigenic potential and invasion capacity of GBM cells in vivo

**Fig. 4:**
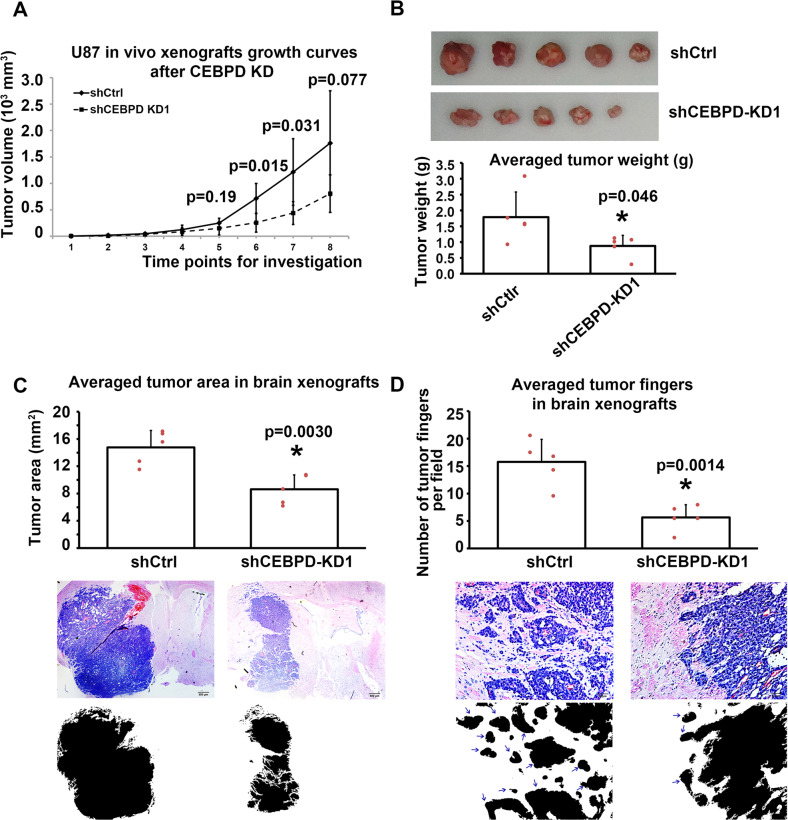
CEBPD knockdown inhibited xenograft tumor growth and invasion capacity of GBM cells in vivo. **A**, **B** Flank xenograft experiments showing the in vivo growth curves of U87 cells in shCtrl (*n* = 5) and shCEBPD groups (*n* = 5). **C** Intracranial xenografts tumor showing the tumor volumes in shCtrl (*n* = 5) and shCEBPD KD (*n* = 5) groups. The bottom binary images showed the tumor areas. **D** The invasion capacity of U87 cells in vivo in shCtrl and shCEBPD KD groups was evaluated by examining the number of tumor fingers in per field. The bottom binary images showed the tumor areas, where islands and protruded areas were indicated by arrows. *p < 0.05, compared with shCtrl group.

The use of CEBPD in the tumorigenesis of GBM cells in vivo was explored, by establishing in vivo flank xenografts in nude mice by implanting U87 cells transfected with shCEBPD-KD1 or shCtrl lentivirus. The in vivo tumor volume growth curves during the 8 weeks of investigation revealed that the mice in the shCtrl group developed significant larger xenografts in later stages compared with the shCEBPD-KD1 group (Fig. 4A). Consistently, at the last investigation time point, the tumor weights in the shCtrl group were significantly greater than those in the shCEBPD-KD1 group (Fig. 4B).

Next, U87 cells infected with shCtrl or shCEBPD-KD1 lentivirus were injected into the brains of nude mice to establish brain xenografts. At the end point, the shCtrl groups developed larger xenografts than the shCEBPD-KD1 groups (Fig. 4C). The invasive capacity of the xenografts was quantitively examined by estimating the relative invasive fingers microscopically by counting the protruded tumor tissue fingers and disseminated areas [44]. As a result, mice brain tumors in the shCEBPD-KD1 group exhibited significantly less invasive fingers compared with the shCtrl group (Fig. 4D). Together with the in vitro study, these results imply that CEBPD expression is important for the invasive capacity of GBM cells.

### CEBPD positively regulated a cohort of proteins involved in the EGFR/PI3K/AKT pathway and ECM function under hypoxic conditions

**Fig. 5:**
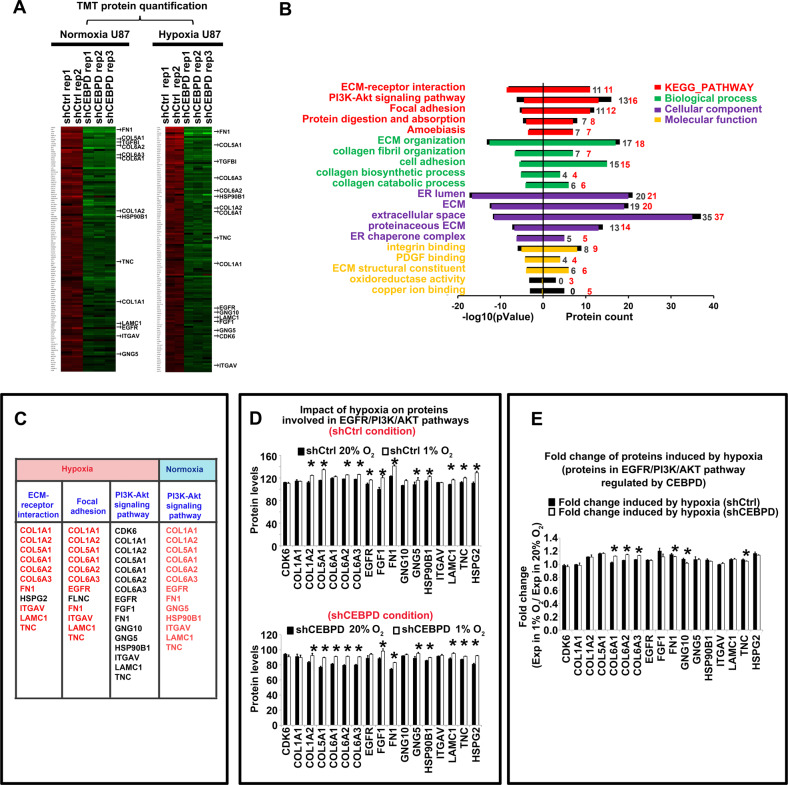
CEBPD-regulated proteins in both normoxia and hypoxia conditions. **A** TMT quantitative proteomic data, showing heatmap of protein levels in shCEBPD KD and shCtrl U87 cells, in both normoxia (20% O_2_) and hypoxia (1% O_2_) conditions. Proteins that are significantly down-regulated at least 1.2-folds after shCEBPD KD were chosen as the CEBPD-regulated proteins. The proteins were sorted in descending order from top to bottom according to their folds changes. **B** KEGG pathway analysis and Gene Ontology (GO) analysis of CEBPD positively regulated proteins in both normoxia and hypoxia conditions in U87 cells. Analyzed GO Categories include Biological process (BP), Cellular component (CM), and Molecular function (MF). The colored bars represent the −log10(*p* value) (left panel) and count of protein (right panel) for each enriched items for CEBPD-regulated protein in normoxia. The black bars, which are covered by the colored bars, represent the corresponding values for CEBPD-regulated proteins in hypoxia. The black parts that are not covered by the colored ones indicated that, for the specific item, the value of the item has a greater value in hypoxia condition than in normoxia condition. The black and red integers in the right panel indicate counts of proteins regulated by CEBPD in normoxia and hypoxia conditions, respectively. **C** Detailed proteins that are belong to the key KEGG pathways affected by CEBPD knockdown in both normoxia and hypoxia conditions. Proteins labeled with red are those which are also belong to the PI3K/Akt signal pathway. **D** Quantitative analysis of hypoxia impact on the protein levels that are belong to the key KEGG pathways, in shCtrl (up panel) or shCEBPD KD (bottom panel) conditions. *p < 0.05, compared with normoxia (20% O_2_) group. **E** Fold changes of hypoxia induced protein levels of the CEBPD-regulated genes in shCtrl and shCEBPD KD groups. *p < 0.05 compared with the shCtrl group. Repeated data for each group were obtained by calculating the ratio of each hypoxia data to the averaged value of normoxia data.

The underlining molecular mechanisms of the CEBPD function were studied. Protein mass spectrometry analysis identified 121 proteins that were significantly down-regulated by at least 1.2-folds after shCEBPD-KD1 knockdown in U87 cells (Fig. 5A). Then, DAVID analysis was performed to decipher the biological functions of these proteins [45]. As a result, the top Kyoto Encyclopedia of Genes and Genomes (KEGG) pathways that were inhibited after CEBPD knockdown included ECM-receptor interaction, focal adhesion, and PI3K-AKT signaling pathways, containing 11, 11, and 13 proteins, respectively (Fig. 5A, B). Notably, almost all the proteins in the ECM-receptor interaction and focal adhesion pathway also belong to the PI3K-AKT signaling pathway (Fig. 5C).

CEBPD-regulated proteins in hypoxia were also analyzed, and 132 proteins were down-regulated by at least 1.2-folds after CEBPD knockdown (Fig. 5A). In total, 99 proteins overlapped in the CEBPD-regulated proteins under normoxic and hypoxic conditions. The top KEGG pathways involving these proteins include ECM-receptor interaction, PI3K-Akt, and focal adhesion pathways (Fig. 5B, C). Interestingly, the value of (−log *p* value) and the number of CEBPD-regulated proteins belong to the PI3K-Akt pathway in hypoxia, are higher than that in normoxic condition (Fig. 5B), indicating the important role of CEBPD for the PI3K-AKT pathway in hypoxia.

In detail, a considerable amount of the CEBPD target proteins were belong to the EGFR/PI3K pathway (Fig. 5C). Interestingly, most of these proteins are ECM molecules and contribute to the invasiveness of glioma cells, including FN1 [46], TNC [47], LAMC1 [48], COL1A1 [19, 48], EGFR [49], CDK6 [50], and COL6A2 [51]. Gene Ontology (GO) analysis also revealed that, the target proteins regulated by CEBPD are significantly enriched in genes for ECM constituent and functions either in normoxic or hypoxic condition (Fig. 5B). Several CEBPD target proteins, whose roles in GBM or invasive capacity have not been clearly studied, were identified, and these proteins include HSP90B1, GNG10, and GNG5.

**Fig. 6:**
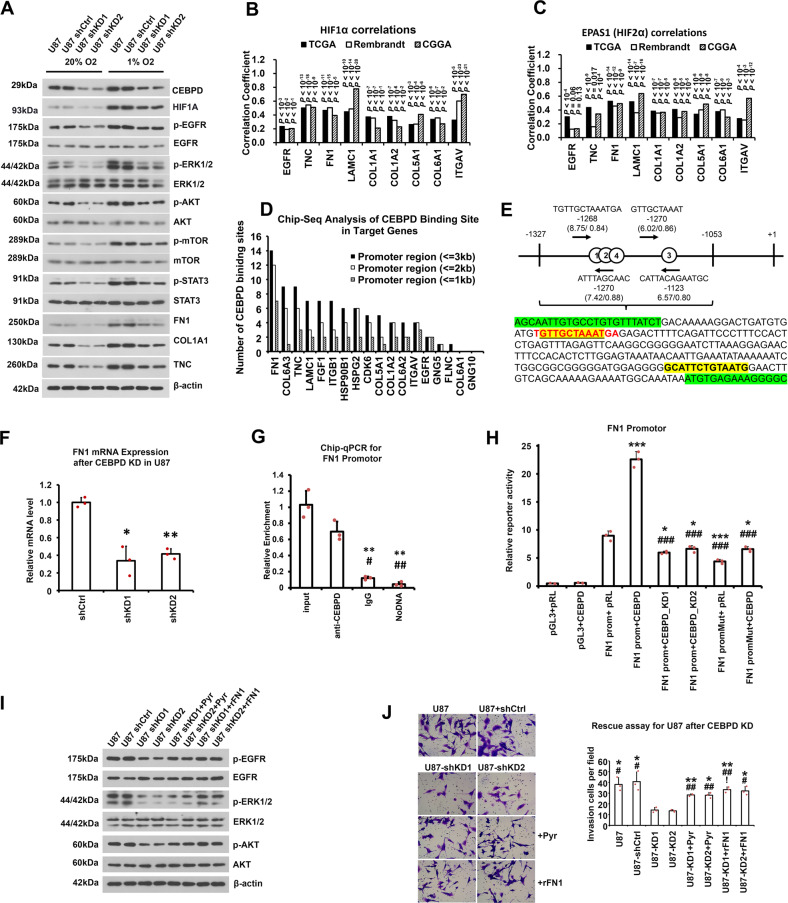
CEBPD positively regulated ECM-mediated EGFR/PI3K pathway activity, and important genes involved in the pathway and invasion capacity, by directly binding to the promotors of key genes in ECM. **A** WB results showing the protein levels of CEBPD and key transducers of the EGFR/PI3K pathway, especially phosphorylated EGFR, ERK1/2, AKT, mTOR, STAT3, and essential genes for the invasion capacity, after CEBPD knockdown in normoxia and hypoxia conditions. **B**, **C** Correlation coefficients and corresponding *p* values (labels above each bar) between HIF1α (**B**) or EPAS1 (**C**) and genes in EGFR/PI3K/Akt signal pathways, which are regulated by CEBPD and contribute to tumor invasion capacity. Pearson correlation was performed between HIF1α or EPAS1and indicated genes labeled in the X axis, in three GBM databases (TCGA, Rembrandt, and CGGA databases). **D** Chip-seq analysis of CEBPD binding sites in CEBPD-regulated proteins belong to the EGFR/PI3K pathway. Bars represented number of CEBPD binding sites identified by Chip-seq in the promoters of the indicated proteins. **E** The DNA fragment of CEBPD binding peaks in the FN1 promoter identified by Chip-seq, which also contained highly reliable CEBPD binding sites as predicted by the JASPAR database [43]. There are 4 highly reliable CEBPD binding consensus sequences (red letters, site 1–4) in the fragment, and the site (1) and (2) had the greatest scores. For each binding site, the sequence, location, and the binding scores (expressed as score/relative score) were labeled; red letters represent positive strand, and yellow shaded letters represent negative strand. Greed shaded letters represent sequences for primers. **F** mRNA levels of FN1 after shCEBPD treatment in U87 cells in normoxia condition. **G** Chip-qPCR showing that CEBPD bind to the FN1 promoter region shown in E in normoxic condition. **H** Luciferase reporter assay showing bind and activation of CEBPD on the promotor of FN1 in normoxia condition. The promoter region of FN1 is shown in (**E**). For mutation analysis, the putative binding sites (site 1 and 2) with the greatest scores were mutated, where the sequence of “TGTTGCTAAATGA” (as shown in **E**) were replaced by “CTGCCGCGGCGGG”. (pGL3: pGL3-Basic vector containing blank control; FN1 prom: pGL3-Basic vector containing FN1 promoter sequence; FN1 promMut: pGL3-Basic vector containing FN1 promoter with mutation; pRL: pRL-TK vector containing blank control; CEBPD: pRL-TK vector containing CEBPD sequence; CEBPD_KD: pRL-TK vector containing CEBPD shRNA sequence.) *p < 0.05, ***p < 0.0001 compared to the FN1 promoter + pRL group; ###*p* < 0.0001 compared to the FN1 promoter + CEBPD group. All of the last 6 groups had *p* < 0.0001 compared to the first 2 groups. **I** WB assays of rescue experiments after CEBPD KD in U87 cells. Protein levels of key transducers of the EGFR/PI3K pathway, including p-EGFR, were examined in U87 cells, U87 cells treated with shCtrl (U87 shCtrl) and shCEBPD lentivirus (U87 shKD1, U87 shKD2), or U87-shCEBPD KD cells which are recused with the α5β1 integrin agonist Pyrintegrin (U87 shKD1+Pyr, U87 shKD2+Pyr) or a recombinated FN1 protein (U87 shKD1 + rFN1, U87 shKD2 + rFN1). The α5β1 integrin is the receptor for FN1. **J** Transwell invasion assays of rescue experiments after CEBPD KD for U87 cells. The cell groups were the same as described in (**I**), and experiments were repeated in triplicate.

The effect of hypoxia on the CEBPD target proteins involved in the EGFR/PI3K pathways was analyzed. Strikingly, most of these proteins are significantly up-regulated in hypoxia (15 out of 17 up-regulated, with 12 of them exhibited *p* < 0.05; Fig. 5D), either in shCtrl or shCEBPD condition. Interestingly, most of the 15 up-regulated proteins in hypoxia exhibited a reduced fold increase in hypoxia after CEBPD knockdown (9 out of 15, with 3 of them exhibited *p* < 0.05; Fig. 5E), including FN1 and TNC (*p* = 0.033, 0.038, respectively, Fig. 5E). In addition, 8 and 10 HRPs (22.22% and 27.78% HRPs) were CEBPD-responsive proteins under normoxic and hypoxic conditions, respectively. Importantly, HIF1A is also a CEBPD-responsive protein based on WB analysis (Fig. 6A). Collectively, these results support that CEBPD played important roles for hypoxia-induced responses, especially for the EGFR/PI3K pathway, ECM function, and invasion capacity.

### CEBPD activates the EGFR/PI3K/AKT pathway through FN1-mediated phosphorylation of EGFR

EGFR/PI3K/AKT pathway is among the core pathways altered in GBM [52] and is important for the invasive capacity of GBM [53]. In addition, EGFR, an important therapeutic target for GBM [16, 17, 49], is among the altered proteins that belong to the PI3K/AKT pathway (Fig. 5D). Therefore, the regulation of EGFR/PI3K activity by CEBPD was further studied.

First, WB assays revealed that CEBPD knockdown reduced the protein levels of phosphorylated/activated form of EGFR, AKT, mTOR, and STAT3, namely p-EGFR, p-AKT, p-mTOR, and p-STAT3 (Fig. 6A, Supplementary Figure S7), either in normoxic or hypoxic condition. Notably, the PI3K/AKT pathway is more activated under hypoxic condition considering that the levels of p-EGFR, p-AKT, p-mTOR, and p-STAT3 were all up-regulated in hypoxia either under shCtrl or shCEBPD-KD condition. Correlation analysis demonstrated that HIF1α and HIF2α were positively correlated with the above genes involved in the EGFR/PI3K/AKT pathway (Fig. 6B, C), confirming that the EGFR/PI3K/AKT pathway is more activated under hypoxic condition. These results strengthened our above conclusion that CEBPD is responsible for the activation of the PI3K/AKT pathway, especially under hypoxic condition.

Second, the genes that contribute to the invasion capacity of GBM cells in the EGFR/PI3K/AKT pathway, including TNC, FN1, and COL1A1, were also down-regulated after CEBPD knockdown in both normoxic and hypoxic conditions (Fig. 6A, Supplementary Figure S7). These genes were also up-regulated under hypoxic condition (Fig. 6A).

The mechanisms of CEBPD-augmented EGFR/PI3K activity were then examined. Firstly, EGFR is a CEBPD target protein in the proteomic results (Fig. 5A), and CEBPD can bind to and activate the EGFR promoter based on Chip-qPCR and luciferase experiments (Supplementary Figure S8). However, WB assays showed that the total EGFR protein level was only marginally reduced after CEBPD knockdown, while the p-EGFR level was significantly reduced (Fig. 6A, Supplementary Figure S7). Therefore, CEBPD promoted the EGFR/PI3K pathway mainly by regulating the phosphorylation but not the transcriptional expression of EGFR. Accordingly, we further deciphered other mechanisms underlining the EGFR/PI3K pathway activation promoted by CEBPD. Considering that ECM-receptor pathway is one of the most apparent KEGG pathways influenced by CEBPD, the associations between them were examined, because interactions between ECMs and their integrin receptors are important for the activation of EGFR/PI3K pathway [54–56].

Chip-seq analysis revealed that the peek binding sites of CEBPD were identified in the promotor region of most of the above proteins, in which the FN1 promotor contained the highest number of CEBPD binding sites (Fig. 6D). Considering that FN1 is among the top proteins that are regulated by CEBPD (Fig. 6A), we then further analyzed whether CEBPD transcriptionally regulated FN1 expression. By comparing the results of predicted TF binding sites from the JASPAR database [43], a peak fragment was identified from the Chip-seq data in the FN1 promotor region, which contained putative CEBPD binding sites with high scores (Fig. 6E). Notably, the mRNA level of FN1 was significantly reduced after CEBPD KD (Fig. 6F), and Chip-qPCR confirmed the binding of CEBPD to this FN1 promoter region (Fig. 6G). Luciferase reporter assay results demonstrated that CEBPD can bind and activate this sequence (Fig. 6H). In addition, the mutation of the putative binding sites 1 and 2 with the greatest scores confirmed the specificity of binding and activation (Fig. 6H).

Interestingly, α5β1 integrin (ITGA5 and ITGB1), the receptor of FN1 (fibronectin) and is a key component in ECM, can activate EGFR phosphorylation and downstream PI3K signals [54]. FN1 and its receptor α5β1 integrin also contribute to the tumorigenesis of glioma [20]. Considering that FN1 is a direct target of CEBPD (Fig. 5A, C; Fig. 6A, D–H), we next investigated whether CEBPD promotes EGFR phosphorylation through the FN1-activated α5β1 integrin. To this, we used α5β1 integrin agonist Pyrintegrin or a recombinant FN1 protein (rFN1) to rescue the α5β1 integrin activity in U87 cells after CEBPD knockdown. As a result, CEBPD knockdown reduced the protein level of p-EGFR but not that of total EGFR, while both Pyrintegrin and rFN1 can rescue the p-EGFR level and PI3K/Akt pathway activity, as indicated by the elevated levels of p-EGFR, p-Akt and p-Erk, which were reduced by CEBPD knockdown (Fig. 6I, Supplementary Figure S9). Transwell invasion assay further demonstrated that both Pyrintegrin and rFN1 can rescue the invasion capacity of U87 cells inhibited by CEBPD knockdown (Fig. 6J).

Overall, CEBPD activated the EGFR/PI3K/AKT pathway and invasion capacity through the ECM (especially FN1)-mediated phosphorylation of EGFR.

### CEBPD remarkably affected the EGFR/PI3K pathway activity under hypoxic condition in GBM patient samples

**Fig. 7:**
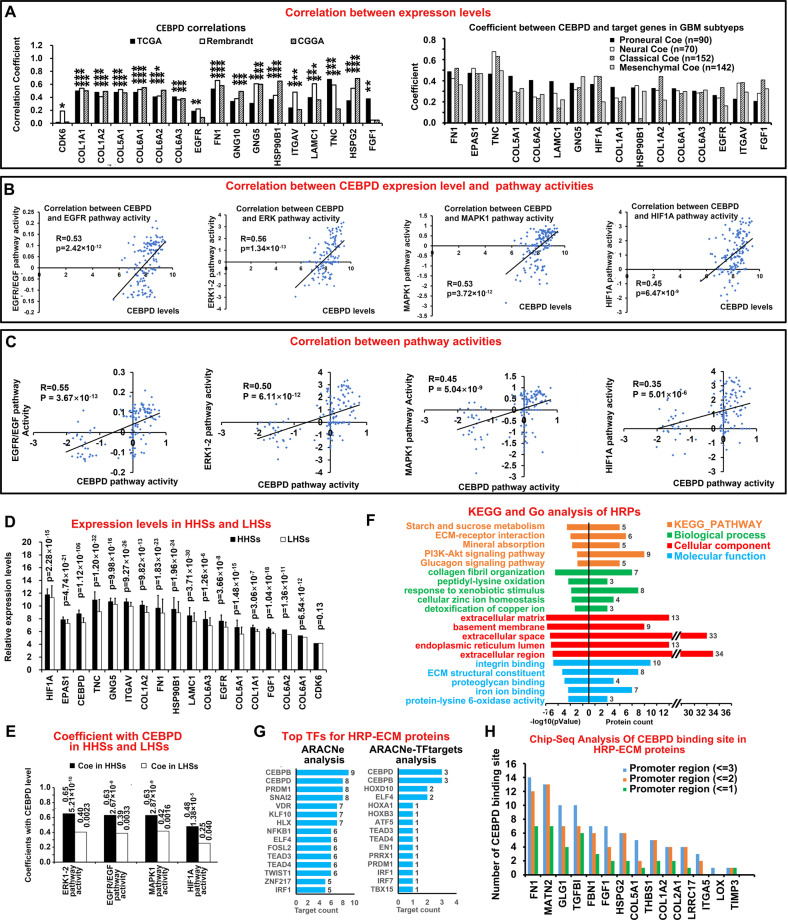
Corroboration of the effect of CEBPD in GBM samples and enrichment analysis of hypoxia-regulated proteins. **A** Correlations between expression levels of CEBPD and genes in EGFR/PI3K pathways (left image) in different databases (TCGA, Rembrandt, and CGGA). *P < 0.05, **(vertical) *P* < 10^−5^, ***(vertical) *P* < 10^−10^. In addition, correlations of CEBPD and these genes in each GBM subtype (Proneural, Neural, Classical, and Mesenchymal) were further performed in the TCGA database to test the impact of subtype on the correlations. **B** Correlations between CEBPD expression level and pathway activities of EGFR/PI3K and HIF1A. **C** Correlations between pathway activities of CEBPD and EGFR/PI3K related pathways, HIF1A pathways. **D** Expression levels of HIFs (HIF1A and EPAS1), CEBPD and genes in EGFR/PI3K pathways in high hypoxic samples (HHSs) and low hypoxic samples (LHSs) in the TCGA database. **E** Coefficients between CEBPD level and EGFR, HIF1A pathway activities in HHSs and LHSs. Digital labels for each bar indicate the coefficients (R, left label) and *p* values (right label). **F** KEGG pathway analysis and Gene Ontology (GO) analysis of HRPs in U87 cells. Analyzed GO Categories include Biological process (BP), Cellular component (CM), and Molecular function (MF). The colored bars represent the −log10(*p* Value) (left panel) and count of protein (right panel) for each enriched items. **G** Top TF analysis of ECM proteins belong to the HRPs (HRP-ECM), as described in Fig. 1. **H** Chip-seq analysis of CEBPD binding sites in HRP-ECM proteins. Bars represented number of CEBPD binding sites identified by Chip-seq in the promoters of the indicated proteins.

Whether the regulatory effects of CEBPD on EGFR/PI3K pathway would be hold in GBM patients need to be considered. Correlation analysis revealed that CEBPD is positively correlated with the expression level of CEBPD target genes that are involved in the EGFR/PI3K/AKT pathway (Fig. 7A), even in each GBM subtype (Proneural, Neural, Classical, and Mesenchymal; Fig. 7A, right panel). In addition, combined with pathway activity analysis [38], we found that both the expression level and pathway activity of CEBPD are positively correlated with the pathway activities of EGFR/PI3K/AKT and HIF1A (Fig. 7B, C). CEBPD also exhibited strong correlation with the pathway activity of itself (Supplementary Fig. S10), thus supporting the reliability of the pathway activity analysis.

The effect of hypoxia on the functions of CEBPD in patients was studied by developing a hypoxia-score for each GBM samples in TCGA datasets. The hypoxia-score for all the TCGA GBM samples were defined by their combined rank index of each gene in the HRGs. Therefore, samples with high hypoxia-scores are more hypoxic than those with lower scores. Then, the GBM samples were split into high-hypoxia samples (HHSs, top 45% samples with hypoxia-score) and low-hypoxia samples (LHS, bottom 45% samples with hypoxia-score). The gene expression levels and pathway activities were analyzed in both HHSs and LHSs.

HIF1A, EPAS1, CEBPD and EGFR were more highly expressed in HHSs than in LHSs (Fig. 7D), indicating the high and low hypoxic features of HHSs and LHSs, respectively. In addition, most of the above genes involved in the EGFR/PI3K/Akt pathway were more highly expressed in HHSs than in LHSs (Fig. 7D), thus confirming that this pathway is more activated under hypoxic condition.

Next, the coefficients between CEBPD and EGFR pathway activities in HHSs and LHSs were calculated. Interestingly, CEBPD exhibited stronger positive correlations with pathway activities of EGFR, ERK1-2, MAPK1, and HIF1A in HHSs than in LHSs (Fig. 7E), indicating that CEBPD remarkably affected these pathways under hypoxic condition.

### ECM-related activities are important components of hypoxia induced responses

Considering that ECM activities are important mediators for CEBPD-induced EGFR/PI3K signal activation, its relationships with hypoxia-induced responses were studied. Interestingly, KEGG and GO analysis revealed that HRPs were enriched in proteins that are involved in ECM-receptor interaction and ECM components (HRP-ECM proteins; Fig. 7F), including essential ECM proteins FN1, collagen family (COL1A2, COL2A1, and COL5A1) and their receptor ITGA5. Interestingly, CEBPD is the top TF that regulates greatest number of HRP-ECM proteins as revealed by the top TF analysis (Fig. 7G). In addition, the promotors of most HRP-ECM proteins contained multiple CEBPD binding peaks based on Chip-seq analysis (Fig. 7H). Therefore, ECM-related activities are important components of hypoxia-induced responses, and CEBPD is a key TF that regulates these activities.

## Discussion

Hypoxia is important therapeutic target for cancer, including GBM [8, 9, 57]. However, considering that hypoxia induces many molecular responses, which form a complex MINW [2], limited is known about the function and structure organization of the HRGs-MINW despite of the availability of a high volume of data about the roles of many single genes, which are actually hub or key genes for the HRGs-MINW. Accordingly, the present study explored and uncovered certain key features of the HRGs-MINW in GBM. In summary, by starting with proteomic technologies, we uncovered that ECM and its activated EGFR/PI3K pathway are important responses induced by hypoxia, and CEBPD is a master TF that regulates these processes. FN1, a key ECM protein, is a direct target of CEBPD mediating these processes. In addition, many other ECM proteins are positively regulated by CEBPD, including TNC, LAMC1, several collagens (COL1A1, COL1A2, COL5A1, COL6A1, COL6A2, and COL6A3) (Fig. 5C) and the subunit of integrin receptors such as ITGAV. Furthermore, most of these genes contained CEBPD binding sites in their promotors (Fig. 6D) and are highly correlated with CEBPD (Fig. 7A), especially TNC and COL6A3. The correlation results are further supported by the correlation analysis in GBM subtypes (Fig. 7A) to exclude the possibility that the correlations were mainly caused by the fact that CEBPD and these genes are up-regulated in mesenchymal subtype. Furthermore, mathematical simulations demonstrated that, if the correlation is mainly caused by different expression level in subtypes, then the correlation in each subtype can be very weak.

Given that ECM-integrin interactions are important outside-in signal pathways, and are promising therapeutic targets [55, 56], our findings uncovered novel up-steam regulatory mechanisms of ECM-integrin interactions. However, the detailed mechanism among these proteins requires future studies. Nevertheless, the finding that CEBPD is a key TF for this process has important theoretical and clinical significance, because ECM-integrin interactions are important activators of the EGFR/PI3K pathway, and the EGFR/PI3K pathway is one of the core pathways altered in GBM [52].

This study was the first reveal that CEBPD is a master TF for hypoxia-induced molecules in both mRNA and protein levels, and this conclusion is consistently obtained from three kinds of independent experimental data (Fig. 1A–C). Although reciprocal regulation is present between CEBPD and HIFs [10, 13, 58], the central role of CEBPD for hypoxia-induced responses uncovered in the present study are not elucidated before. The mechanism behind the central role of CEBPD is that it regulates ECM-integrin proteins, while ECM-integrin interactions are also key components of hypoxia induced responses (Fig. 7F–H). As a hypoxia-induced protein, CEBPD was identified in the list of PANSGs, but not in HRPs and SR-HRGs, because for HRPs, CEBPD is not covered in the proteomic panel in the high-throughput quantification. For SR-HRGs, CEBPD is overlapped in 2 sets out of the 5 sets, while we used genes overlapped in at least 3 sets as our SR-HRGs. Notably, for PANSGs analysis, CEBPD is specifically highly expressed in PAN area (Fig. 2I), consistent with previous report [14].

A notable novel finding of the present study is that FN1 is a direct target of CEBPD. FN1 is also up-regulated by hypoxia (Fig. 5A, D, E, Fig. 6A), consistent with other studies. Nevertheless, FN1 is induced by TGFβ signal [59], rather than by HIFs [60]. Consistently, HIF binding elements were not identified in FN1 promotor in GBM cells when we checked the previously reported Chip-seq studies [61, 62]. Therefore, it is more possible that FN1 up-regulation in hypoxia is mediated through HIF-independent pathways such as TGFβ signal or as reported in present study, by CEBPD. TGFβ signal pathway is closely associated with ECM-integrin outside-in signals [55, 56, 63], and TGFβ signal pathway is activated in hypoxia [6, 59].

Moreover, the critical proteins of TGFβ signal pathway, TGFBI and a component of integrin receptor ITGA5 were up-regulated under hypoxic condition (TGFBI and ITGA5 were up-regulated by 1.11 and 1.12 folds in hypoxia, *p* < 0.05). TGFBI is important for the activation of TGFβ signal pathway in GBM [64, 65], which can activate integrins [60]. Notably, TGFBI and its receptor subunit ITGAV are potential direct targets of CEBPD supported by the following information: 1. TGFBI and ITGAV proteins are significantly down-regulated after CEBPD KD (Fig. 5A; TGFBI data were not included in Fig. 5C–E, it was down-regulated by 1.49 folds after CEBPD KD, *p* = 2.10 × 10^−5^). 2. Many CEBPD binding elements are present in the promotor of TGFBI (Fig. 7H) and ITGAV (Fig. 6D). 3. CEBPD exhibited highly positive correlation with TGFBI and ITGAV (Fig. 7A; the coefficients between TGFBI and CEBPD were 0.58, 0.54, and 0.62 in TCGA, REMBRANDT, and CGGA GBM datasets, respectively). Therefore, one important potential down-stream results of CEBPD expression in hypoxia is the activation of TGFβ signal pathway by directly regulating TGFBI and ITGAV. In addition, this pathway may also contribute to the activation of FN1 [59, 60]. Therefore, CEBPD-induced FN1 expression can activate the EGFR/PI3K pathway by promoting EGFR phosphorylation. However, CEBPD may also induce FN1 up-regulation and the down-stream pathways through other indirect ways, such as through TGFβ signal-induced FN1 expression. Detailed investigation of the interplays among CEBPD, TGFβ signal pathway, ECM and hypoxia will be the focus of future studies.

One of the key signal feature of GBM is the activation of receptor tyrosine kinase (RTK), especially the EGFR-related activation of PI3K/AKT pathways [52]. Alterations in canonical PI3K/MAPK pathways are identified in more than 90% GBM samples [52, 66], and the most common and important gene alteration in this pathway is EGFR, whose amplification or mutation is identified in more than half (57%) of GBM samples [66]. Specifically, EGFR is responsible for cancer cell survival and invasiveness under hypoxic condition [67]. However, treatment strategies that target EGFR have thus far failed in clinical trials [17]. Therefore, the mechanisms underlying sustained EGFR pathway activation have been important issues for long time and need to be further explored [16]. In the present study, the EGFR/PI3K/Akt pathway activation was increased under hypoxic condition, and is dependent on CEBPD expression and its induction of ECM-integrin activation, especially direct transcriptional regulation of FN1. In terms of mechanisms, the activation of this pathway is mainly dependent on the phosphorylation rather than the transcriptional regulation of EGFR. Other possible downstream targets of CEBPD may contribute to the activation of the PI3K/AKT pathway, such as FGF1 (Fig. 5A, C), or other unidentified growth factors. The hypoxia-induced activation of EGFR/PI3K pathway activity in the present study (Fig. 6A) is consistent with previous reports [68]. Notably, EGFR activation also promoted HIF-induced signaling [69, 70], indicating a more complicated network interaction underlining EGFR/PI3K pathway activation in GBM. In addition, EGFR activity can promote CEBPD expression [71], implying a positive feedback between them, and targeting CEBPD would be promising strategies to break the signal loops. Collectively, our data deepens the understanding of the activation mechanisms of EGFR/PI3K pathways, especially in hypoxia, and provide novel potential therapeutic avenues to target this pathway.

## Conclusion

Our study revealed novel features of the HRGs-MINW, in which CEBPD functions as a key TF controlling hypoxia-induced tumorigenic potential of GBM. This process mainly occurs through ECM-integrin signal-mediated EGFR phosphorylation and subsequent activation of EGFR/PI3K pathway, which ultimately increases the invasion capacity of GBM. FN1, a key ECM protein, is a direct transcriptional target of CEBPD that mediates these processes.

## Supplementary information

Supplementary Figures S1-S7

Supplementary Table S1

Supplementary Table S2

Supplementary Table S3

Supplementary Tables

Original Data File

The reproducibility checklist
