# Supplementary material for: CEBPD is a master transcriptional factor for hypoxia regulated proteins in glioblastoma and augments hypoxia induced invasion through extracellular matrix-integrin mediated EGFR/PI3K pathway
Source: Cell Death Dis. 2023 Apr 14;14(4):269. doi: 10.1038/s41419-023-05788-y (PMC10104878; doi:10.1038/s41419-023-05788-y)
Supplement: Supplementary file 1 — Supplementary Figures S1-S7 [file 41419_2023_5788_MOESM1_ESM.docx]

**Supplementary figures**


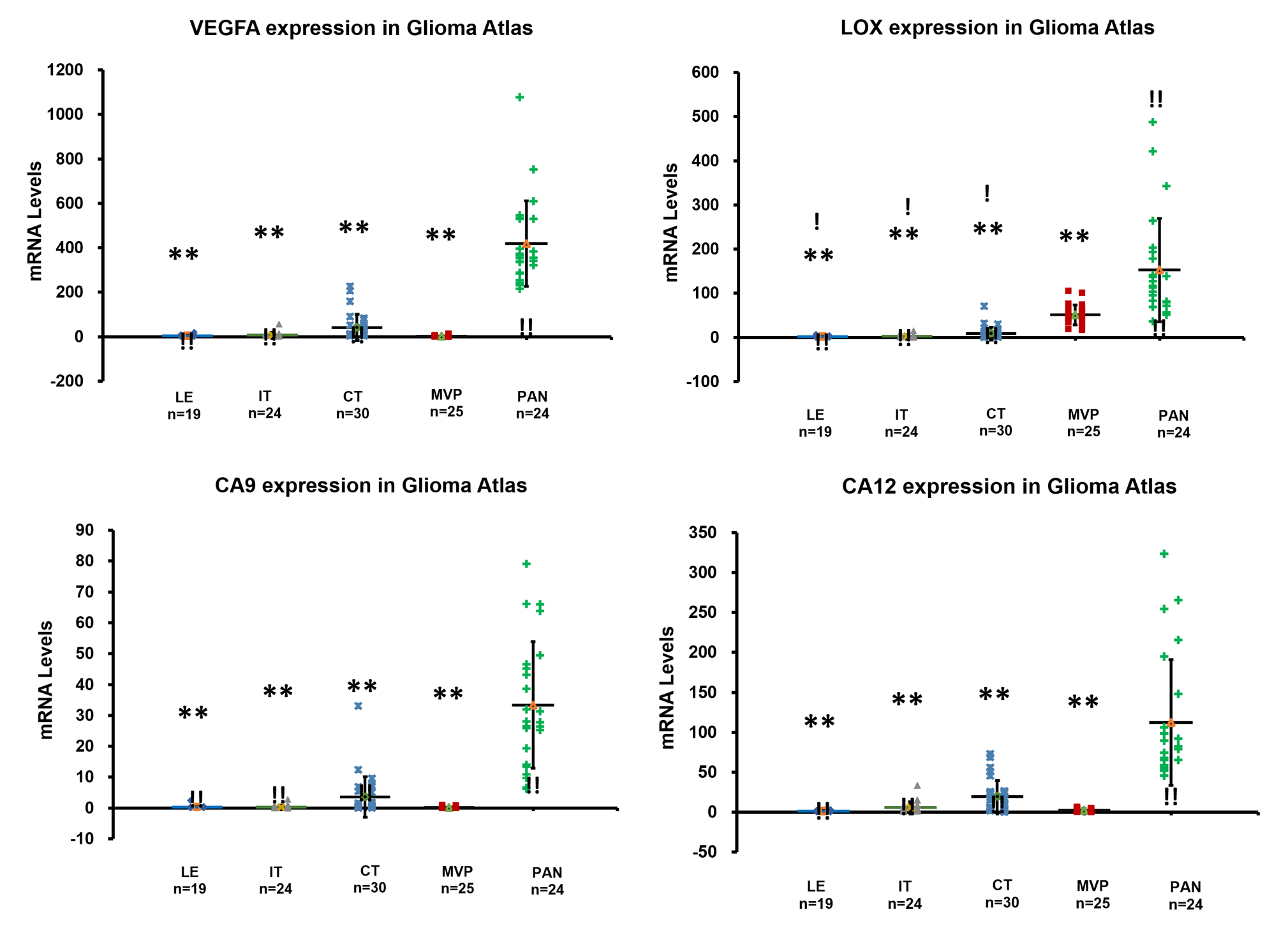


Supplementary Figure S1. Expression levels of hypoxic marker genes in different areas in GBM tissues, including leading edge (LE), infiltrating tumor (IT), cellular tumor (CT), pseudopalisading cells around necrosis (PAN), and microvascular proliferation (MVP) areas, in the glioblastoma atlas data. * p <0.05, ** p < 0.001, compared to PAN; ! p <0.05, !! p < 0.001, compared to MVP.


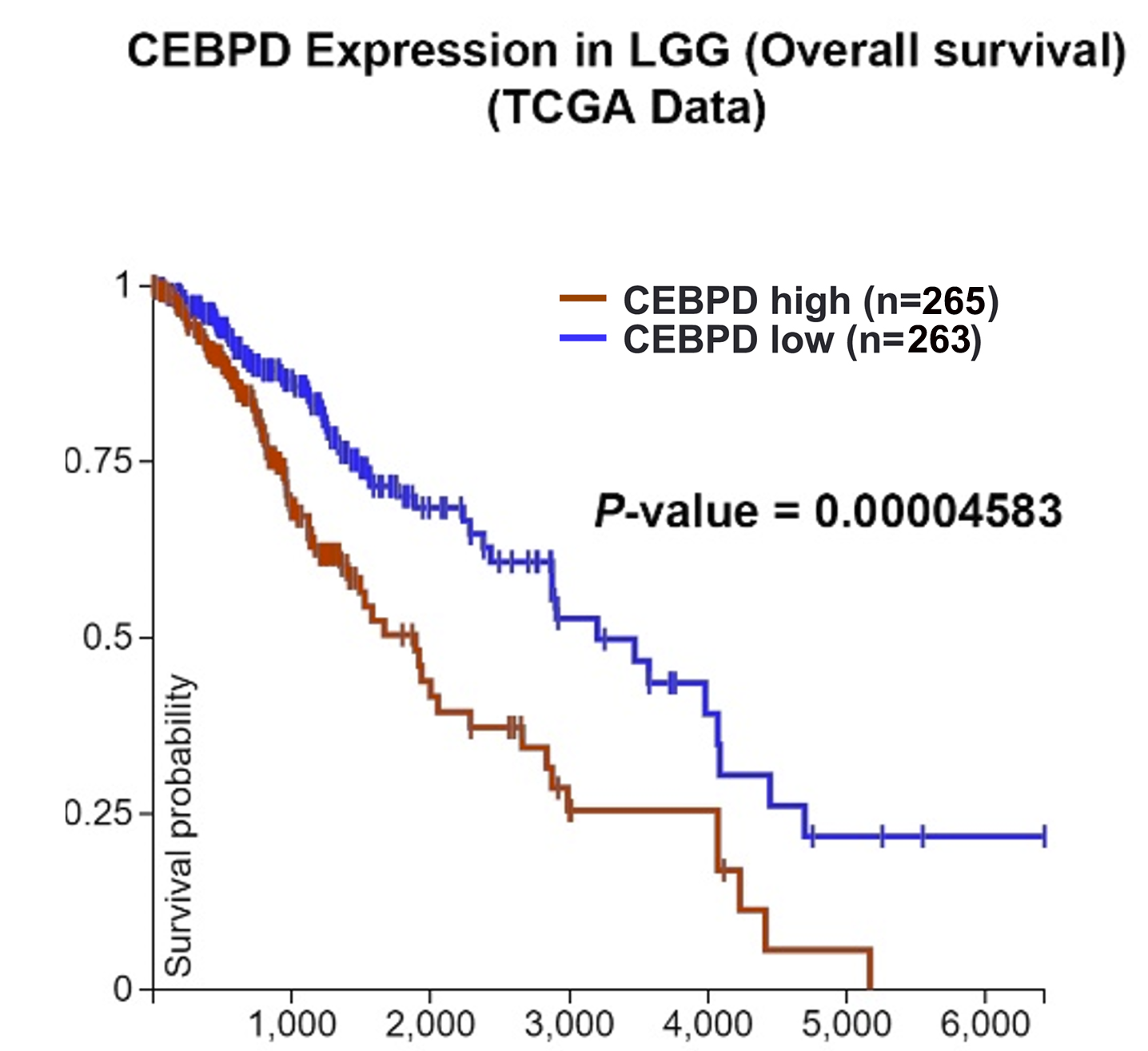


Supplementary Figure S2. Survival analysis in TCGA LGG data showing high expression of CEBPD predict poor prognosis of LGG patients.


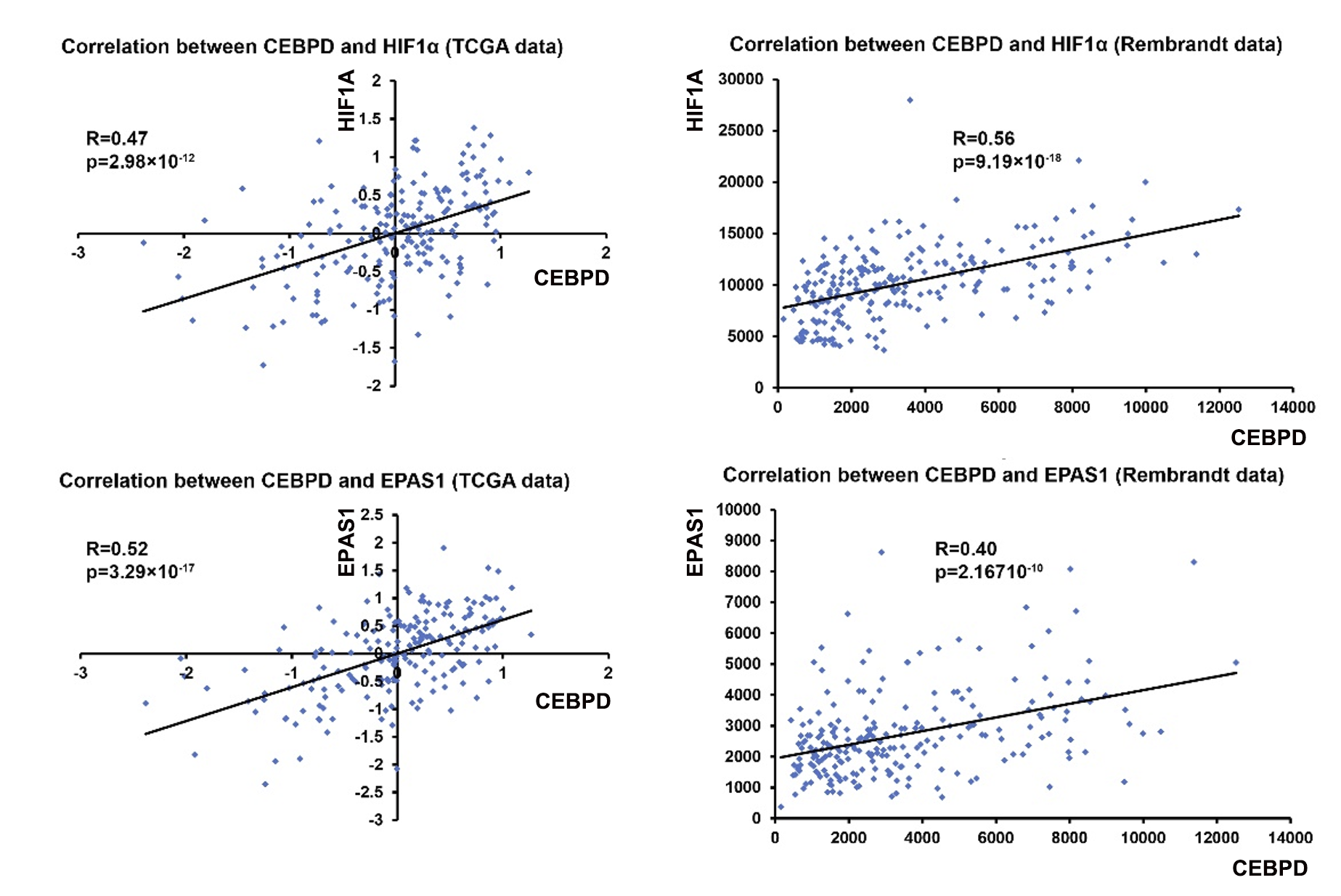


Supplementary Figure S3. Correlations of CEBPD with HIF1α and HIF2α (EPAS1) in TCGA and Rembrandt GBM databases.


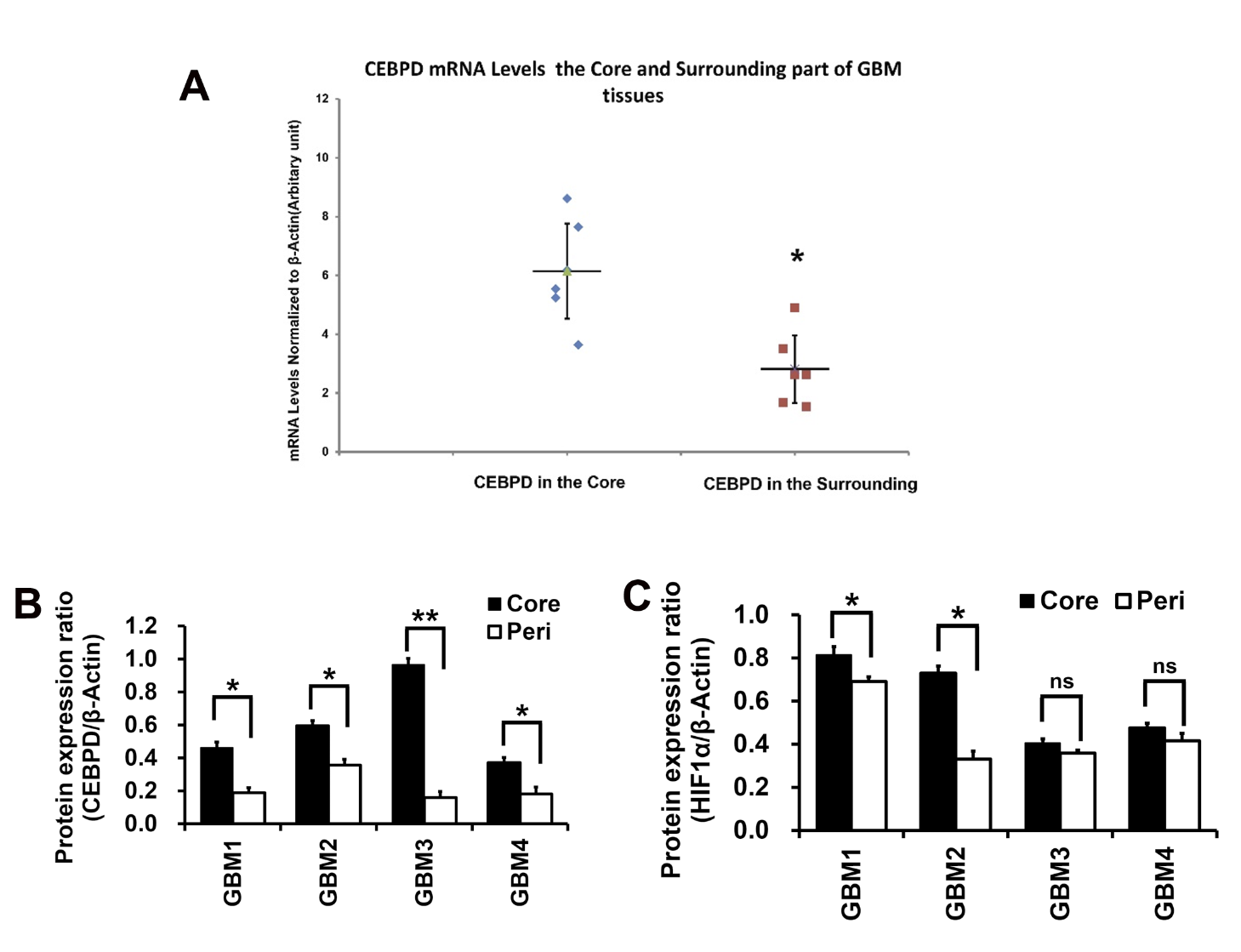


Supplementary Figure S4. (A) Expression of CEBPD mRNA levels in core and peripheral regions of GBM patient samples. (* p < 0.05); (B) and (C) WB quantification of CEBPD (B) and HIF1A (C) proteins levels (n = 3 independent experiments), relating to Fig. 2G.


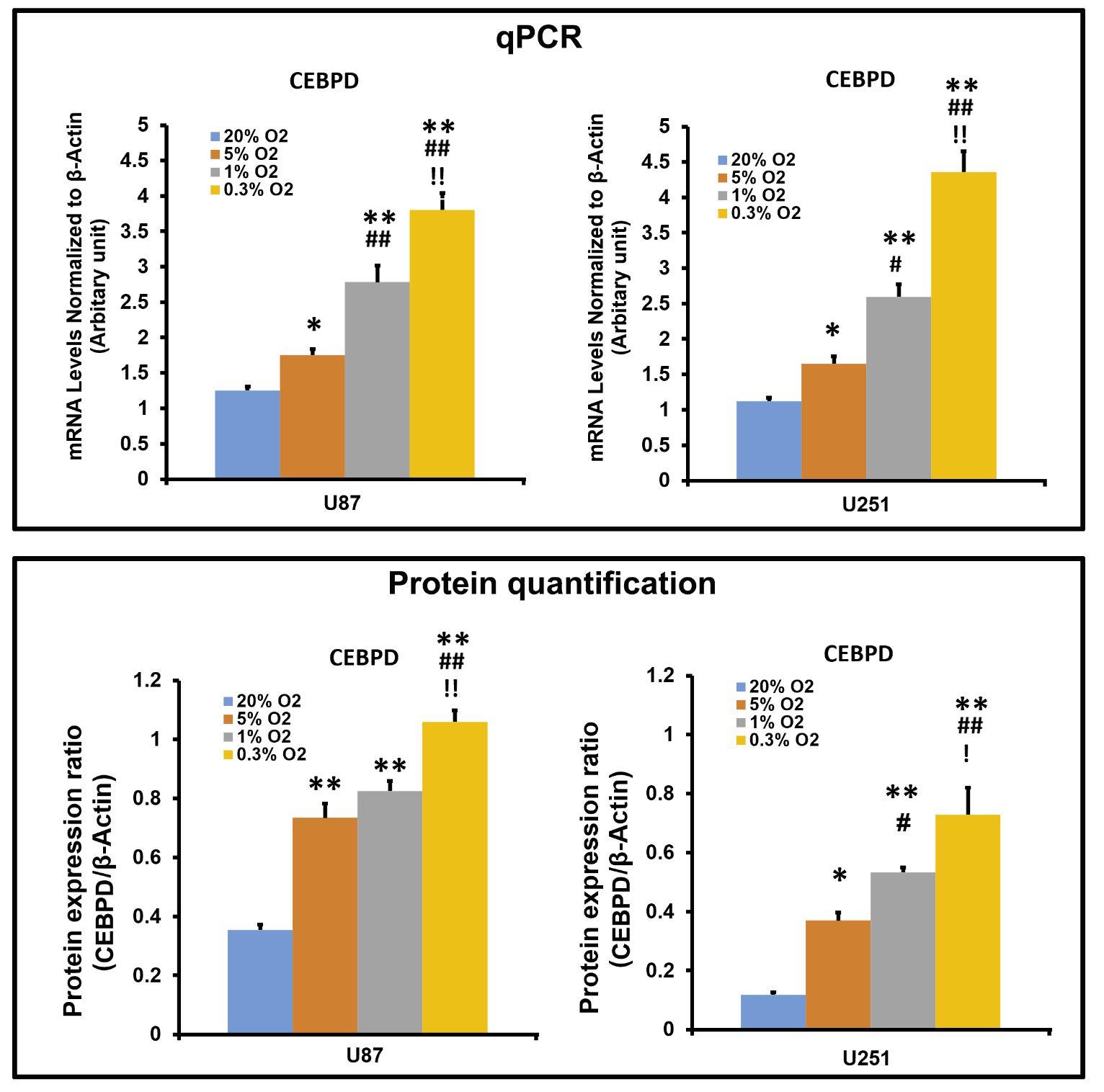


Supplementary Figure S5. (A) qPCR results showing the mRNA levels of CEBPD in U87 and U251 GBM cells in hypoxia condition. (B) WB quantification of CEBPD proteins levels in different oxygen level conditions (n = 3 independent experiments), relating to Fig. 2J. * p < 0.05, ** p<0.001 compared to 20% O2; # p < 0.05, ## p<0.001 compared to 5% O2; ! p < 0.05, !! p<0.001 compared to 1% O2.


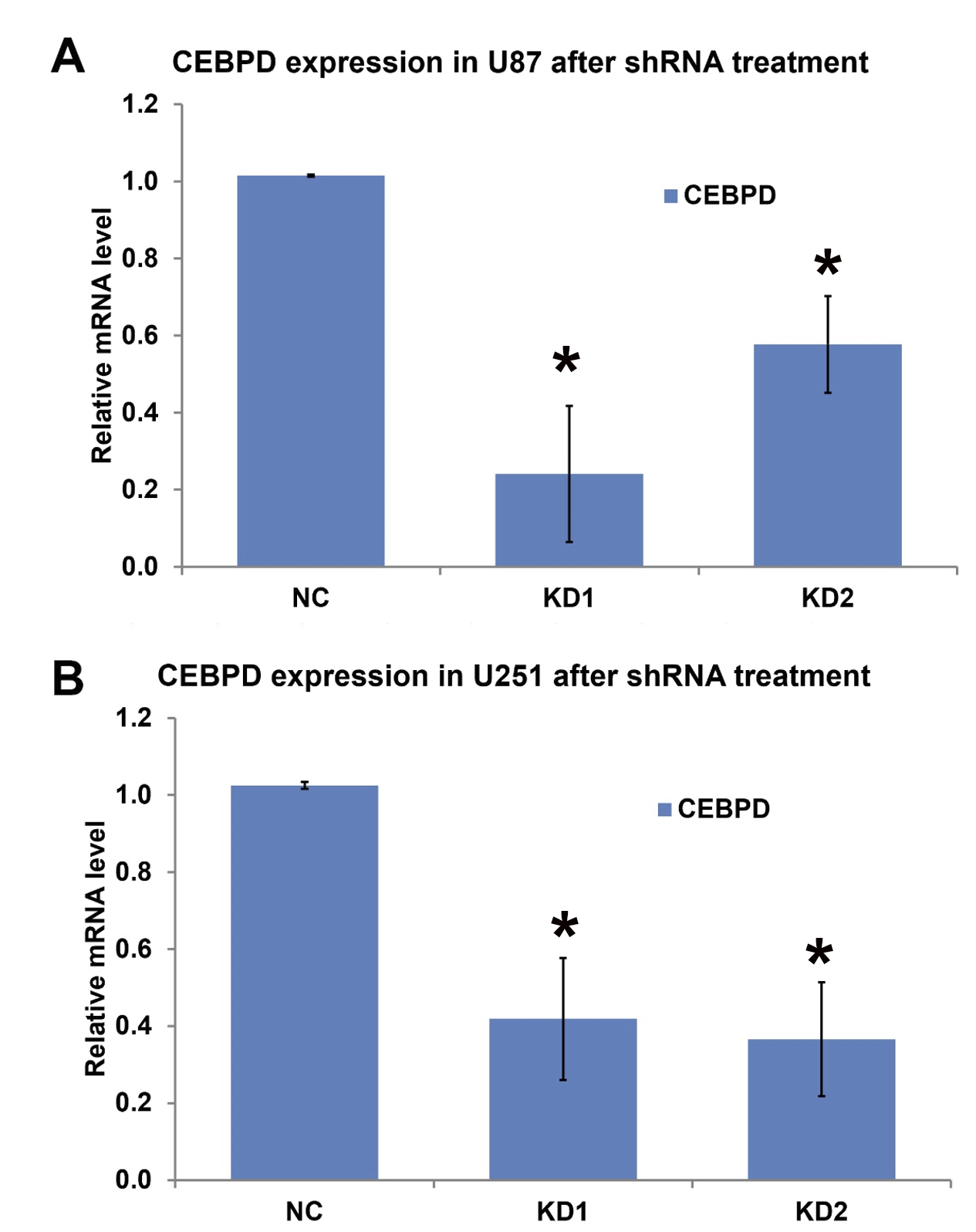


Supplementary Figure S6. qPCR results showing the knockdown efficiency of shCEBPD KD1 and KD2 in U87 and U251 cells. * p <0.05 compared to NC group.


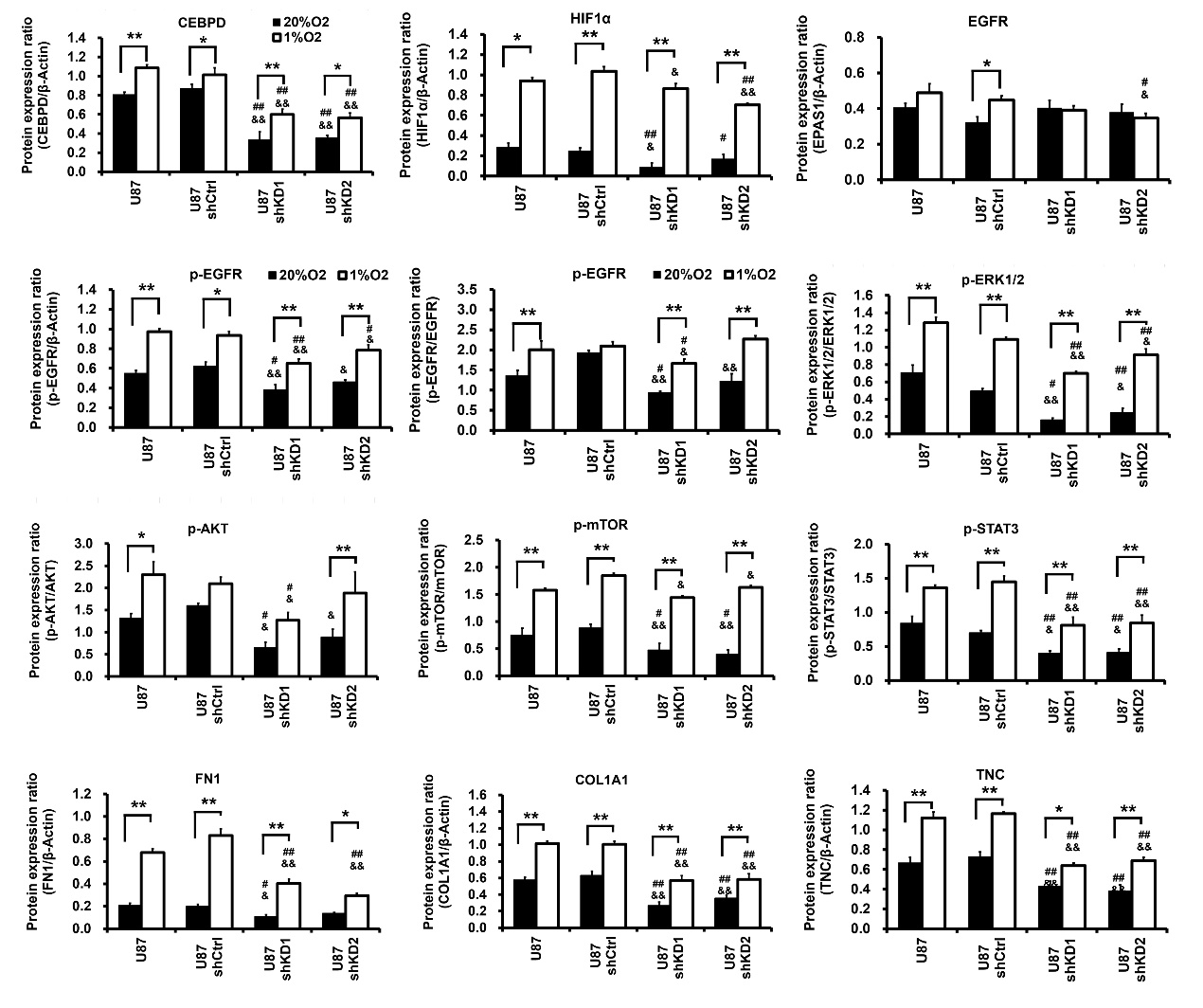


Supplementary Figure S7. WB quantification of proteins levels (n = 3 independent experiments), relating to Fig. 6A. ** p<0.001, * p <0.05 compared between paired hypoxia and normoxia groups; ## p <0.001, # p < 0.05 compared to corresponding parent U87 cells (hypoxia or normoxia); && p <0.001, & p < 0.05 compared to corresponding U87 shCtrl cells (hypoxia or normoxia). Note: p-EGFR normalized to either β-Actin or total EGFR were performed.


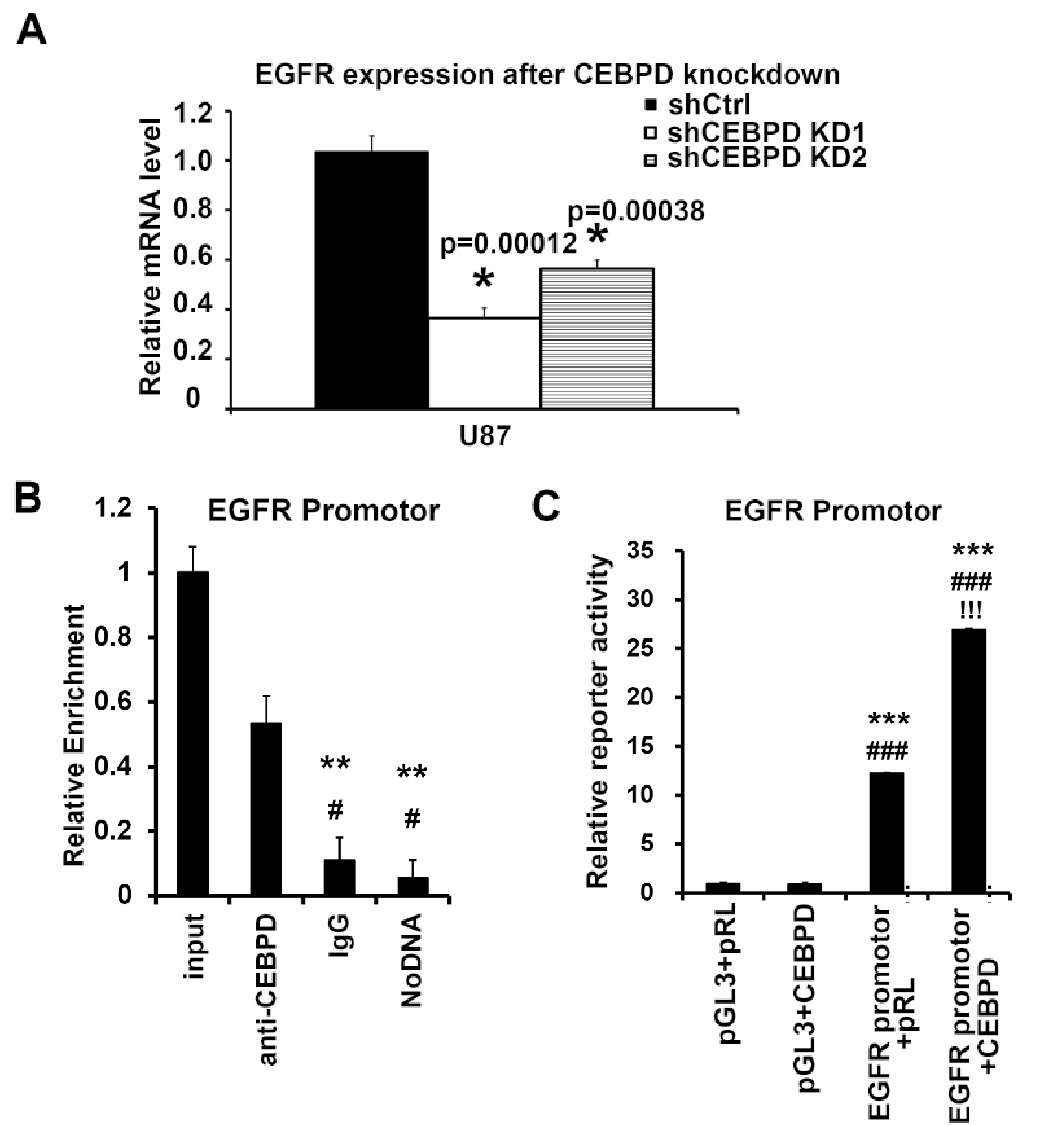


Supplementary Figure S8. CEBPD positively regulated EGFR/PI3K pathway activity. (A) CEBPD knockdown inhibited the mRNA levels of EGFR; * p <0.05 compared to shCtrl. (B) Chip-qPCR results showing that CEBPD binds directly to the promotor region of EGFR; ** p < 0.01 compared to input group; # p < 0.05 compared to anti-CEBPD group. (C) Luciferase reporter assay showing that CEBPD activates the promotor of EGFR; *** p < 0.0001 compared to pGL3+pRL group;


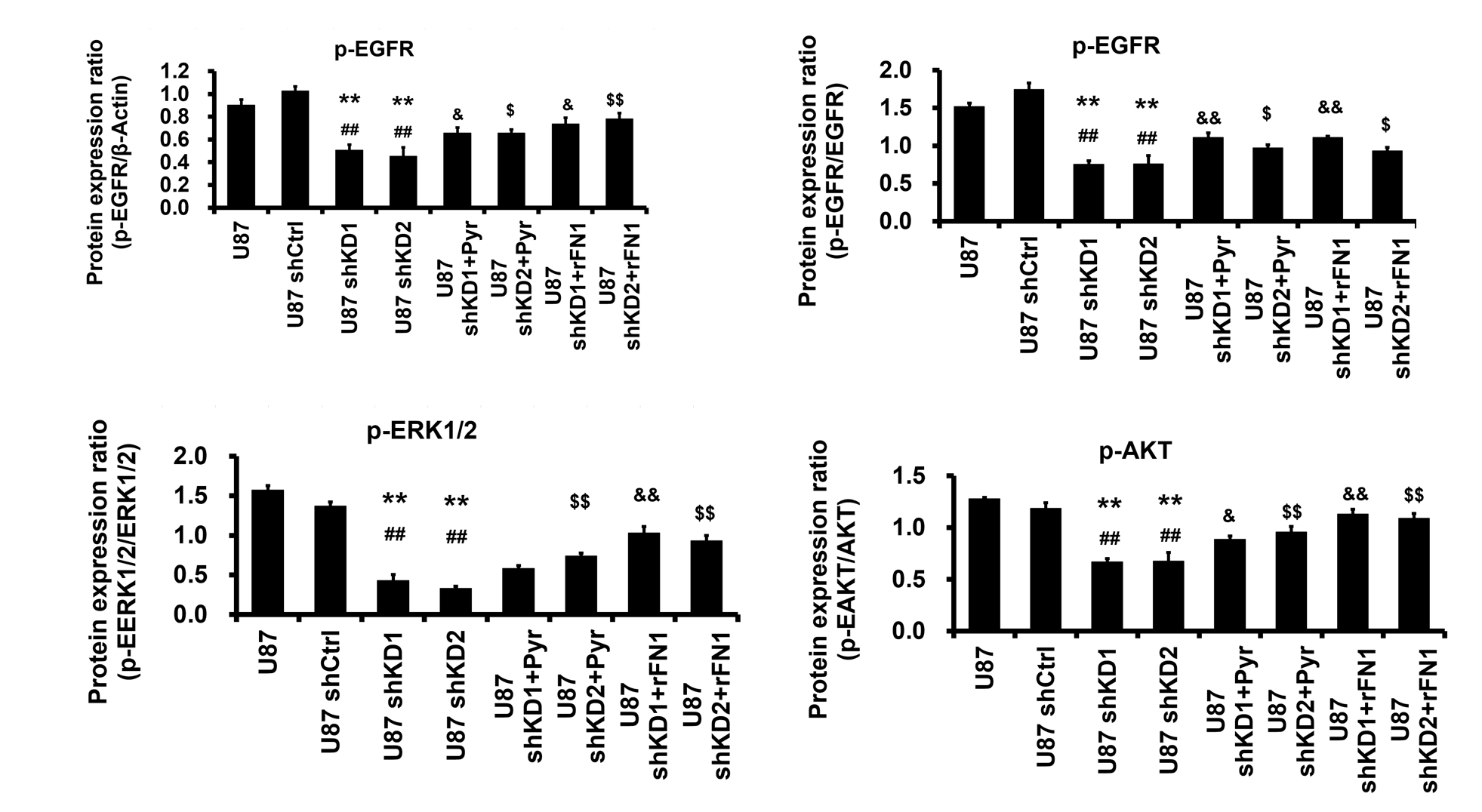


Supplementary Figure S9. WB quantification of proteins levels (n = 3 independent experiments), relating to Fig. 6I. ** p<0.001, * p <0.05 compared to parent U87 cells; ## p <0.001, # p < 0.05 compared to U87 shCtrl cells; && p<0.001, & p <0.05 compared to U87 shKD1cells; $$ p<0.001, $ p <0.05 compared to U87 shKD2cells. Note: p-EGFR normalized to either β-Actin or total EGFR were performed.


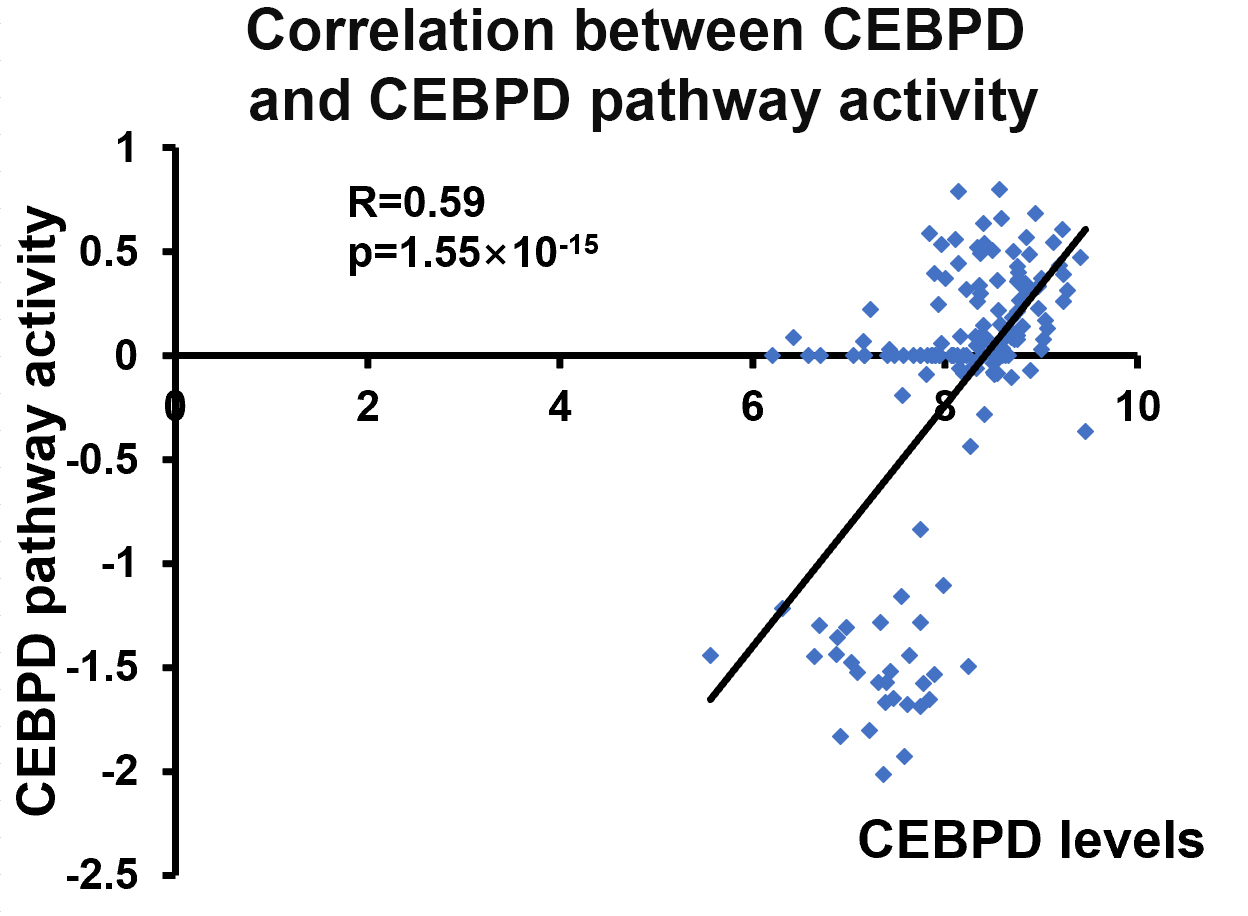


Supplementary Figure S10. Correlation between CEBPD expression levels and CEBPD pathway activities in TCGA GBM database.
