# Supplementary material for: CEBPD is a master transcriptional factor for hypoxia regulated proteins in glioblastoma and augments hypoxia induced invasion through extracellular matrix-integrin mediated EGFR/PI3K pathway
Source: Cell Death Dis. 2023 Apr 14;14(4):269. doi: 10.1038/s41419-023-05788-y (PMC10104878; doi:10.1038/s41419-023-05788-y)
Supplement: Supplementary file 5 — Supplementary Tables [file 41419_2023_5788_MOESM5_ESM.docx]

Supplementary Tables

Supplementary Table S1

List of hypoxia regulated proteins

Supplementary Table S2

All human TF targets reconstructed by ARACNe algorithm

Supplementary Table S3

Human TF targets by combining the ARACNe algorithm and experimentally based TF targets (ARACNe_hTFtarget_combined)

Supplementary Table S4

Multivariate survival analysis including CEBPD expression in Rembrandt data of GBM patients.

Supplementary Table S4

Multivariate survival analysis in Rembrandt data indicated that high expression of CEBPD is a negative factor for survival of GBM patients.

|  | B | SE | Wald | df | Sig. | Exp(B) | Lower | Upper |
| --- | --- | --- | --- | --- | --- | --- | --- | --- |
| Age | 0.025 | 0.013 | 3.569 | 1 | 0.059 | 1.026 | 0.999 | 1.053 |
| Gender | 0.349 | 0.317 | 1.213 | 1 | 0.271 | 1.417 | 0.762 | 2.637 |
| KPS | -0.028 | 0.007 | 15.916 | 1 | 0.00 | 0.972 | 0.959 | 0.986 |
| CEBPD | 0.452 | 0.209 | 4.683 | 1 | 0.03 | 1.571 | 1.044 | 2.366 |
