# Supplementary figures and images for: CEBPD is a master transcriptional factor for hypoxia regulated proteins in glioblastoma and augments hypoxia induced invasion through extracellular matrix-integrin mediated EGFR/PI3K pathway
Source: Cell Death Dis. 2023 Apr 14;14(4):269. doi: 10.1038/s41419-023-05788-y (PMC10104878; doi:10.1038/s41419-023-05788-y)

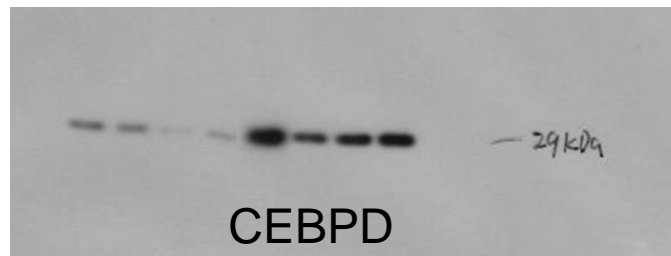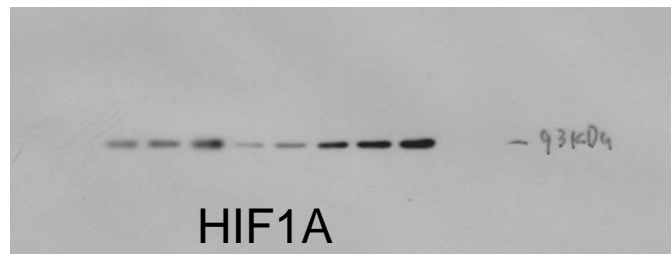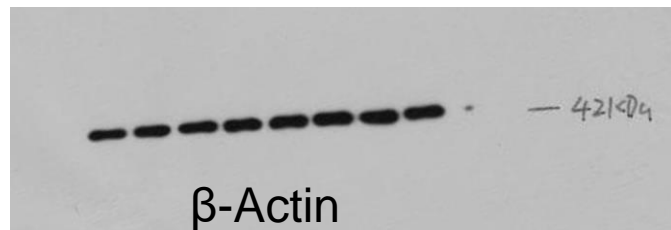

Fig. 2C

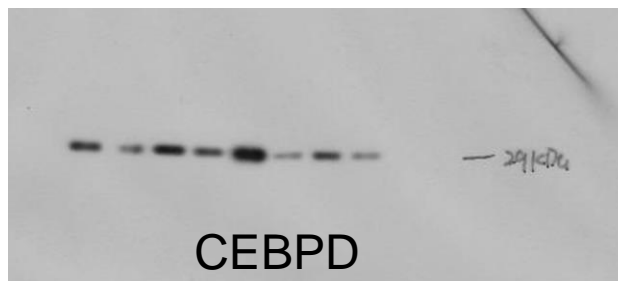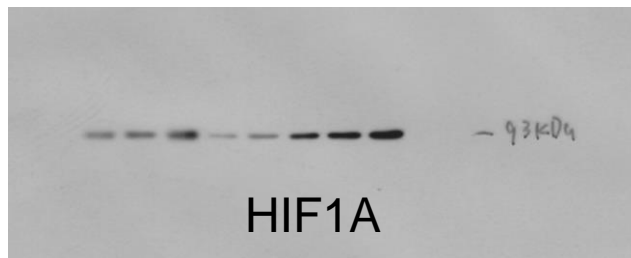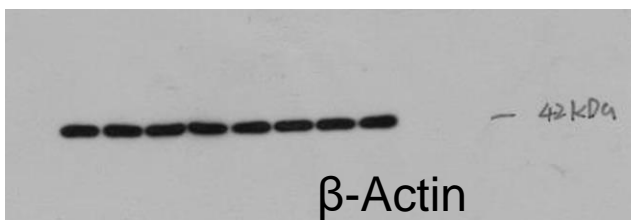

Fig. 2G

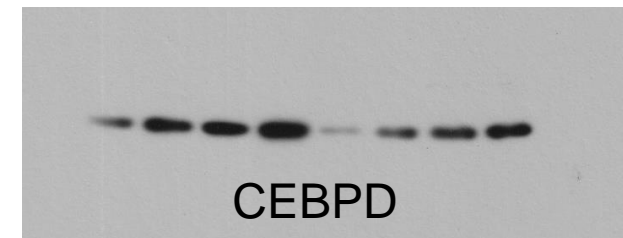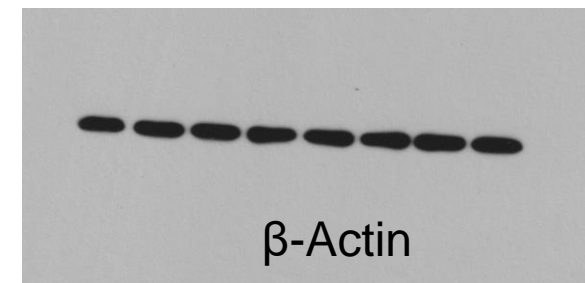

Fig. 2J

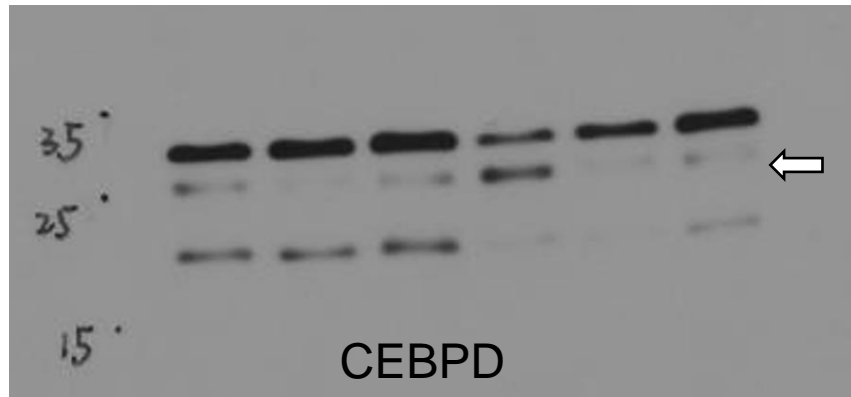

Fig. 3A, U87

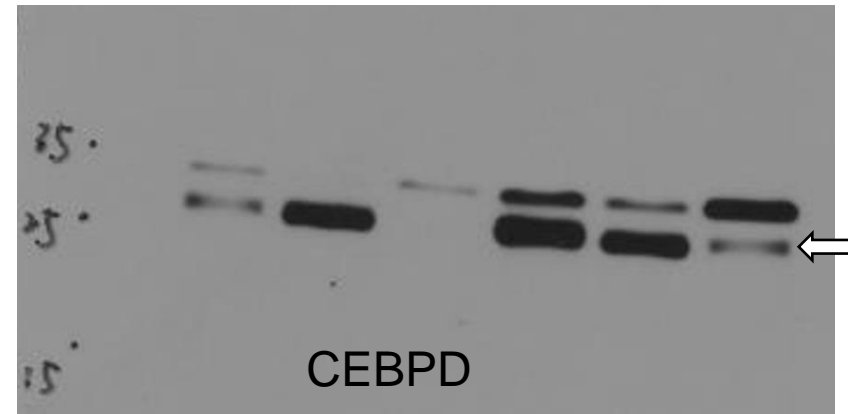

Fig. 3A, U251

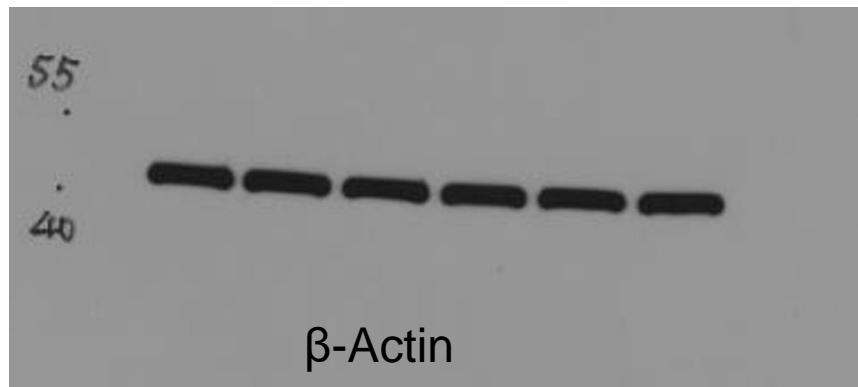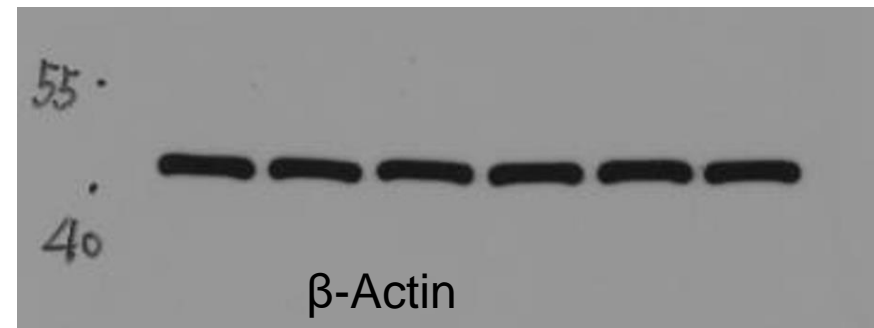

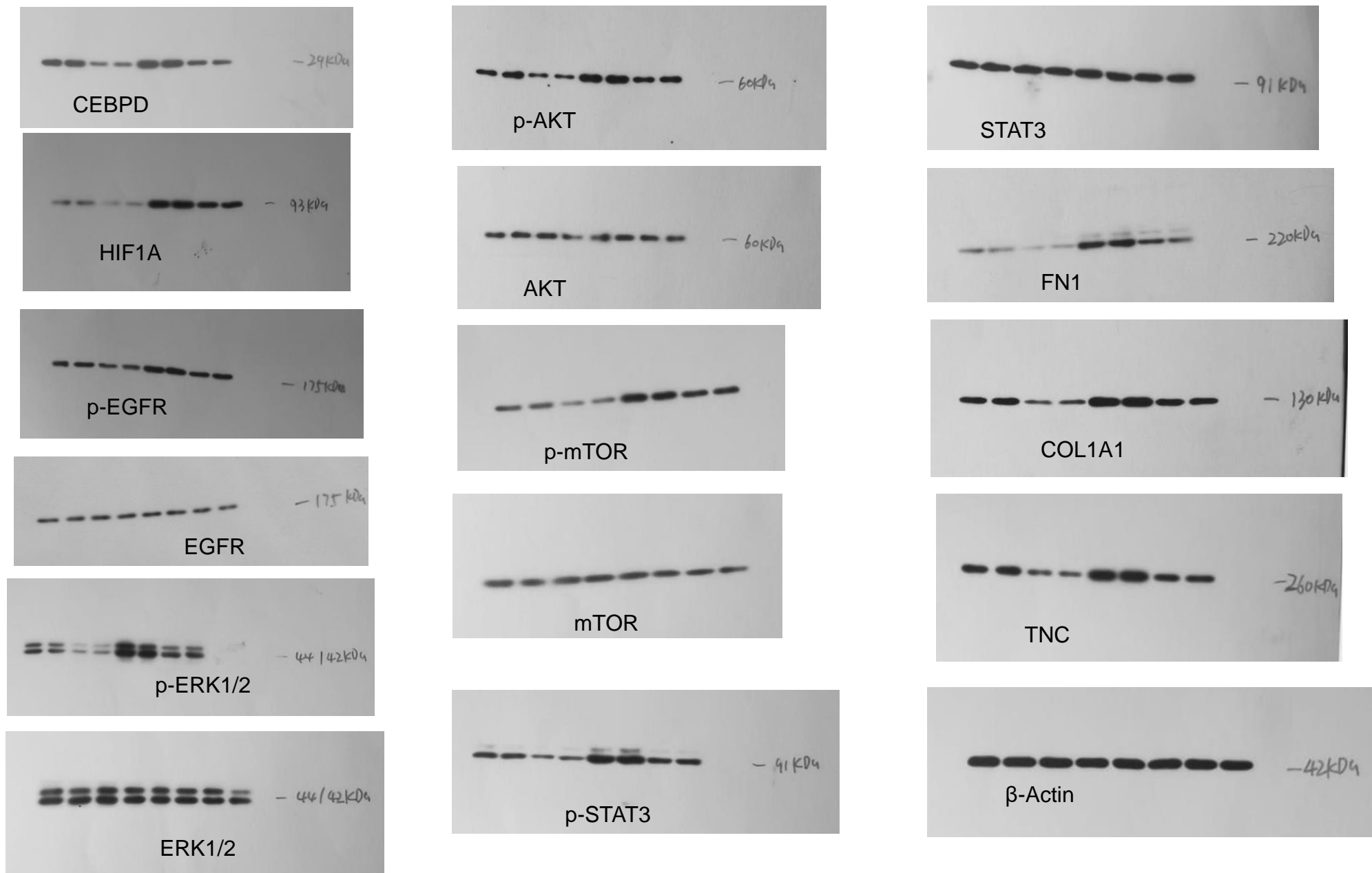

Fig. 6A

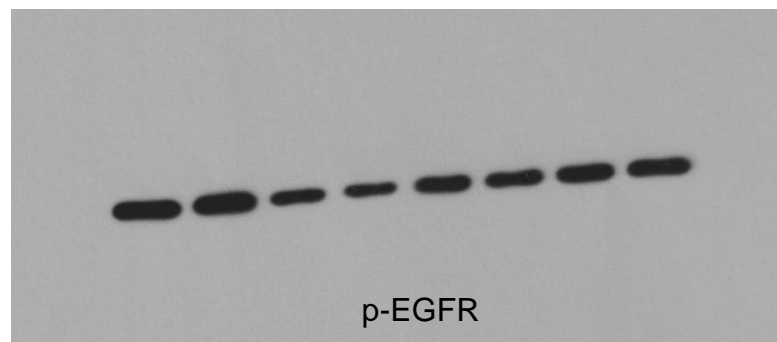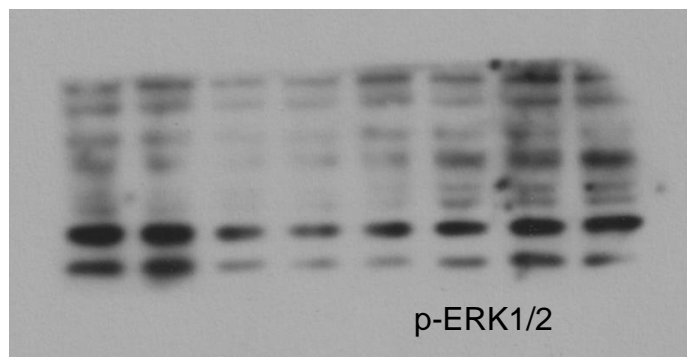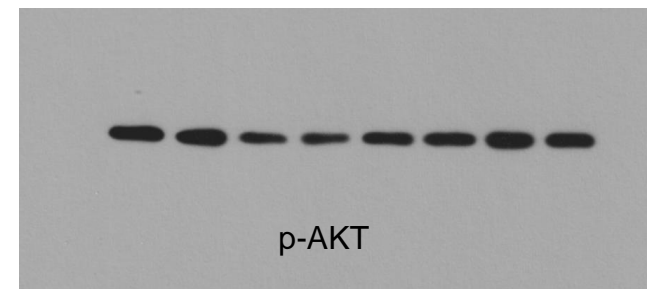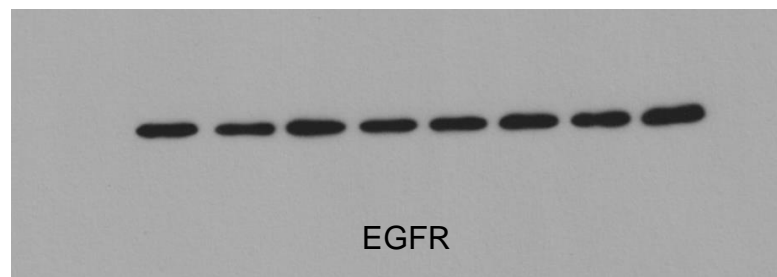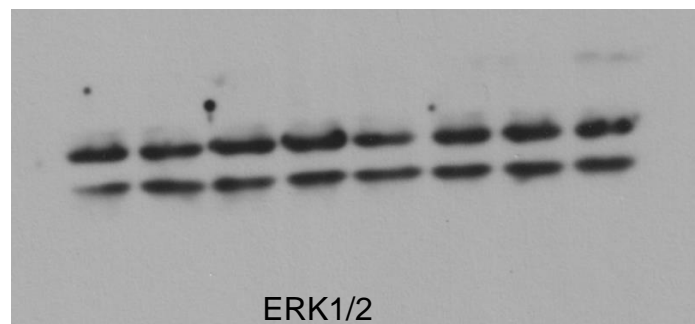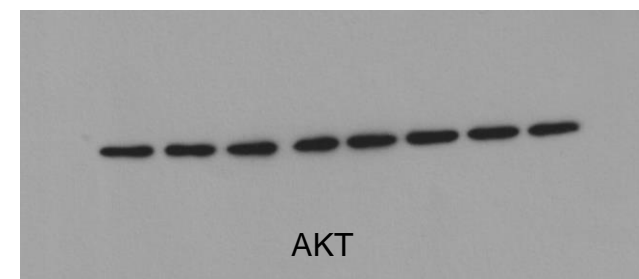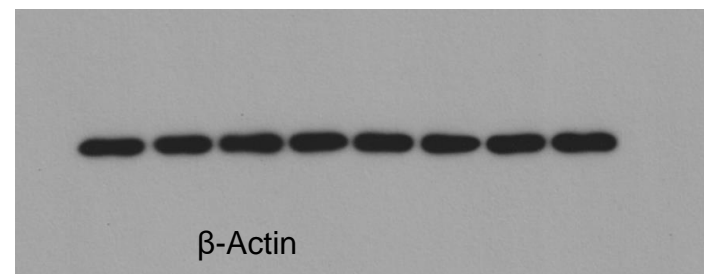

Fig. 6l

Supplement: Supplementary file 6 — Original Data File [file 41419_2023_5788_MOESM6_ESM.pdf]
